# Sex differences in resting skeletal muscle and the acute and long-term response to endurance exercise in individuals with overweight and obesity

**DOI:** 10.1016/j.molmet.2025.102185

**Published:** 2025-06-16

**Authors:** Simon I. Dreher, Thomas Goj, Christine von Toerne, Miriam Hoene, Martin Irmler, Meriem Ouni, Markus Jähnert, Johannes Beckers, Martin Hrabě de Angelis, Andreas Peter, Anja Moller, Andreas L. Birkenfeld, Annette Schürmann, Stefanie M. Hauck, Cora Weigert

**Affiliations:** 1 Institute for Clinical Chemistry and Pathobiochemistry, Department for Diagnostic Laboratory Medicine, University Hospital Tübingen, Tübingen, Germany; 2 Institute for Diabetes Research and Metabolic Diseases of the Helmholtz Zentrum München at the University of Tübingen, Tübingen, Germany; 3 German Center for Diabetes Research (DZD e.V.), München-Neuherberg, Germany; 4 Metabolomics and Proteomics Core, Helmholtz Center Munich, German Research Center for Environmental Health, Neuherberg, 85764, Germany; 5 Institute of Experimental Genetics, Helmholtz Zentrum München, Neuherberg, Germany; 6 Department of Experimental Diabetology, German Institute of Human Nutrition Potsdam-Rehbruecke (DIfE), Nuthetal, Potsdam, Germany; 7 School of Life Sciences, Chair of Experimental Genetics, Technical University Munich, Freising, 85764, Germany; 8 Department of Internal Medicine IV, University Hospital Tübingen, Germany; 9 Institute of Nutritional Science, University of Potsdam, Brandenburg, Germany

**Keywords:** Skeletal muscle, Sex-specific, LC-MSMS based quantitative proteomics, Exercise

## Abstract

**Objectives:**

Endurance exercise reduces the risk of metabolic diseases by improving skeletal muscle metabolism, particularly in individuals with overweight and obesity. As biological sex impacts glucose and fatty acid handling in skeletal muscle, we hypothesized sex differences at the transcriptomic and proteomic level in the acute response to exercise and after an 8-week exercise intervention.

**Methods:**

We analyzed skeletal muscle biopsies from 25 sedentary subjects (16f/9 m) with overweight and obesity using transcriptomics and proteomics at baseline, after acute exercise, and following an 8-week endurance training program. Regulation of sex-specific differences was studied in primary myotubes from the donors.

**Results:**

At baseline, differentially methylated CpG-sites potentially explain up to 59% of transcriptomic and 67% of proteomic sex differences. Differences were dominated by higher abundance of fast-twitch fiber type proteins, and transcripts and proteins regulating glycogen degradation and glycolysis in males. Females showed higher abundance of proteins regulating fatty acid uptake and storage. Acute exercise induced stress-responsive transcripts and serum myoglobin predominantly in males. Both sexes adapted to 8-week endurance training by upregulating mitochondrial proteins involved in TCA cycle, oxidative phosphorylation, and β-oxidation. Training equalized fast-twitch fiber type protein levels, mainly by reducing them in males. In vivo sex differences in autosomal genes were poorly retained in myotubes but partially restored by sex hormone treatment.

**Conclusions:**

Our findings highlight sex-specific molecular signatures that reflect known differences in glucose and lipid metabolism between female and male skeletal muscle. After just 8 weeks of endurance training, these sex differences were attenuated, suggesting a convergence towards a shared beneficial adaptation at the molecular level.

## Highlights

• Sex differences in transcripts and proteins are deeply rooted in the epigenome.
• Proteomic sex differences in skeletal muscle reflect fiber-type dimorphism.
• Males showed higher muscular stress markers after the initial acute endurance exercise.
• Eight weeks of endurance training attenuates the sex differences in the proteome.

## Graphical abstract

**Figure:**
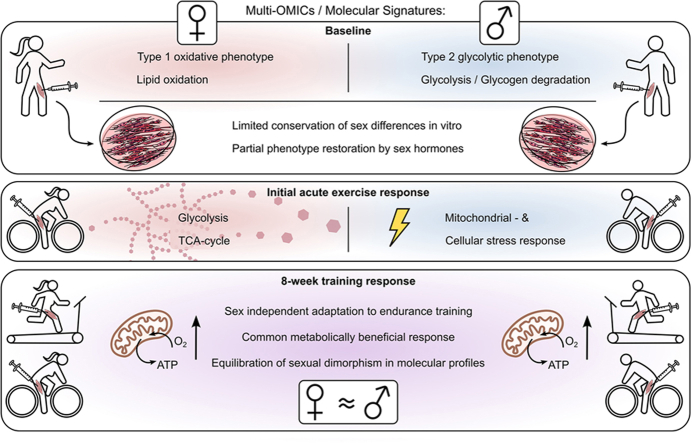

## 1 Introduction

The skeletal muscle comprises 35–45% of total body weight in healthy non-obese females and males and is of significant importance for the regulation of glucose and fatty acid metabolism. It is responsible for 85% of insulin-dependent glucose uptake, which can increase up to 50-fold during intense muscular work [1]. The impact of biological sex on the skeletal muscle metabolism in health and disease, including physical activity and prevention of type 2 diabetes, has been neglected for decades. This perception is undergoing significant changes, since it has been recognized that the biological sex has a clear impact on the regulation of peripheral metabolism and insulin sensitivity [2], with implications for the pathophysiology and clinical manifestation of metabolic diseases and response to treatment [3,4]. Hyperinsulinemic-euglycemic clamp data which consider differences in body weight or lean mass indicate higher glucose disposal in women, driven by higher insulin-induced glucose uptake in skeletal muscle [[5], [6], [7]]. Several studies also reported greater clearance of triglycerides from VLDL in women [8,9], driven by greater triglyceride extraction by skeletal muscle [10]. Together with the higher plasma fatty acid availability for skeletal muscle repeatedly reported in women [11], this may cause the higher intramyocellular lipid content in female skeletal muscle [12,13]. Notably, not only intramyocellular triglyceride (IMTG) storage but also IMTG lipolysis is suggested to be higher in women than in men [14].

Beyond that, sex as a biological variable influences exercise metabolism and potentially the adaptation to regular training. It has been reported that during exercise, females utilize more fat, and males more carbohydrates [[15], [16], [17]]. In contrast, after exercise, females showed elevated glucose oxidation and males elevated lipid oxidation [11,18]. The response to training may also differ since compared to females, males were reported to have greater increase in VO_2_peak, muscle mass and strength in response to exercise despite already higher levels at baseline [19,20].

Exercise is a major contributor to the reduction in the risk of developing metabolic disease, however the effect of sex on the transcriptome and proteome response to exercise is incompletely understood. Investigations in the field reported transcriptional and epigenetic differences [[21], [22], [23]], or compared the skeletal muscle proteome of both sexes in an untrained and a trained group [24].

In our study, we provide an in-depth multi-omics analysis of molecular differences between female and male skeletal muscle in response to exercise. We investigated skeletal muscle biopsies of individuals with overweight and obesity obtained during an 8-week endurance training intervention [25,26], as this group is at increased risk of developing glucose intolerance and dysregulated lipid metabolism. We applied a multi-omics approach employing DNA methylation, transcriptomics and proteomics. Together with the differences at baseline, we studied also sex-based differences in the transcriptomic and proteomic response to acute exercise and after the 8-week endurance training. Moreover, we investigated whether sex-specific differences were conserved in the myotubes generated from the myogenic satellite cells of the same donors and whether treatment with sex hormones can mimic a sex-specific transcriptional regulation identified *in vivo*.

## 2 Methods

### 2.1 Study participants

Study design and participants were described previously [25]. Inclusion criteria for the study were sedentary lifestyle (<120 min of physical activity per week, questionnaire-based), BMI >27 kg/m^2^, but absence of manifest diabetes. The age range of male participants was 28–46 years. The age range of female participants was 19–59 years, including one postmenopausal individual. Two women used oral contraceptives. The influence of menstrual cycle phase was not controlled. However, none of these participants appeared as outliers in PCA of the multi-omics datasets. Baseline muscle biopsies and training was not synchronized with the menstrual cycle. All participants gave written informed consent and the study protocol was approved by the ethics committee of the University of Tübingen and was in accordance with the declaration of Helsinki. The study was registered at Clinicaltrials.gov as trial number NCT03151590.

### 2.2 Study design

Participants performed 1 h of supervised endurance training three times per week for 8 weeks, consisting of 30 min of cycling on an ergometer and 30 min of walking on a treadmill. Before and after the training period, all participants underwent maximal spiroergometry as an incremental cycling test using an electromagnetically braked bicycle ergometer, to determine the individual VO_2_peak. The test was terminated at volitional exhaustion or muscular fatigue. Peak VO_2_ was defined as the mean VO_2_ over the last 20 s before the cessation of exercise and was assessed by metabolic gas analysis. The training intensity was individually set at 80% of the VO_2_peak determined before the intervention and was not changed throughout the training period. Training intensity was controlled by heart rate based on predetermined 80% of the VO_2_peak and individually set. For anthropometric and clinical data of the included 16 females and 9 males, see Table 1, Table 2. Body fat mass and distribution were measured by magnetic resonance imaging [27]. Metabolic and fitness parameters were assessed as described in [25]. The insulin sensitivity index was estimated from the oGTT by the method of Matsuda and DeFronzo (ISI_Mats_) [28]. Not considering sex-specific differences, methylome and transcriptome data from this cohort were reported recently [29].

**Table 1:** Anthropometric data of study participants.

	Baseline			Trained			Fold change		
	Female	Male	*p*-Value	Female	Male	*p*-Value	Female	Male	*p*-Value
**Sex**	16	9							
**Height [cm]**	164 ± 4.13	181 ± 6.23	**<0.001**						
**Weight [kg]**	86.4 ± 14.6	101 ± 13.8	**0.020**						
**Age [Years]**	27.9 ± 8.79	33.2 ± 5.59	**0.005**						
**BMI**	31.9 ± 4.37	30.6 ± 3.63	0.479	31.7 ± 4.45	30.2 ± 3.92	0.350	0.99 ± 0.02	0.99 ± 0.02	0.567
**Waist to hip**	0.86 ± 0.04	0.94 ± 0.04	**0.001**	0.86 ± 0.05	0.93 ± 0.04	**0.001**	1 ± 0.02	0.99 ± 0.02	0.404
**TAT [L]**	42.4 ± 11.0	35.4 ± 9.17	**0.044**	41.7 ± 11.1	34.2 ± 8.95	**0.039**	0.98 ± 0.03	0.97 ± 0.05	0.508
**SCAT [L]**	16.4 ± 5.47	13.0 ± 4.58	**0.044**	15.8 ± 5.85	12.4 ± 4.30	0.075	0.96 ± 0.06	0.95 ± 0.07	0.819
**VAT [L]**	2.69 ± 1.14	4.72 ± 1.42	**0.004**	2.62 ± 1.14	4.42 ± 1.36	**0.006**	0.98 ± 0.16	0.94 ± 0.05	0.296
**AT-L.Ex [L]**	17.9 ± 4.00	13.1 ± 3.53	**0.008**	17.9 ± 3.86	12.5 ± 3.42	**0.003**	1 ± 0.05	0.95 ± 0.03	**0.010**
**AT femur [L]**	516 ± 104	361 ± 77.7	**0.001**	495 ± 109	350 ± 68.7	**0.003**	0.96 ± 0.08	0.98 ± 0.07	0.932
**NAT-L.Ex [L]**	15.5 ± 2.16	22.0 ± 3.00	**<0.001**	16.0 ± 1.93	22.1 ± 2.99	**<0.001**	1.03 ± 0.04	1.01 ± 0.02	**0.048**
**IAT/BW**	1.06 ± 0.23	1.18 ± 0.13	0.135	1.28 ± 0.27	1.42 ± 0.19	0.152	1.21 ± 0.12	1.21 ± 0.12	0.994
**Systolic BP [mm Hg]**	130 ± 11.6	139 ± 14.8	0.184	124 ± 12.9	141 ± 12.2	**0.007**	0.96 ± 0.13	1.02 ± 0.13	0.107
**Diastolic BP [mm Hg]**	87.6 ± 9.00	89.8 ± 15.3	0.722	80.2 ± 11.4	89.2 ± 7.88	**0.038**	0.92 ± 0.12	1.02 ± 0.16	0.154
**Glucose fast. [mmol/L]**	5.01 ± 0.33	5.28 ± 0.40	0.121	4.87 ± 0.28	5.28 ± 0.37	**0.016**	0.97 ± 0.05	1 ± 0.06	0.274
**Glucose 120 [mmol/L]**	5.65 ± 1.23	5.73 ± 1.13	0.879	5.33 ± 0.89	5.84 ± 2.23	0.932	0.97 ± 0.2	1.06 ± 0.49	0.821
**Insulin fast. [pmol/L]**	110 ± 44.5	95.6 ± 34.4	0.396	106 ± 35.8	89.7 ± 35.5	0.307	1.06 ± 0.38	0.98 ± 0.28	0.561
**Insulin 120 [pmol/L]**	626 ± 463	401 ± 186	0.533	496 ± 322	387 ± 359	0.322	0.85 ± 0.32	1.11 ± 1.2	0.671
**ISI_MATS_**	8.22 ± 3.38	9.58 ± 6.80	0.799	9.00 ± 4.10	9.63 ± 5.02	0.755	1.14 ± 0.37	1.1 ± 0.24	0.843
**HbA1C [mmol/mol]**	33.9 ± 2.64	35.0 ± 2.16	0.286	33.4 ± 2.42	33.8 ± 2.70	0.753	0.99 ± 0.05	0.97 ± 0.05	0.451
**HbA1C [%]**	5.25 ± 0.24	5.35 ± 0.20	0.286	5.21 ± 0.22	5.24 ± 0.25	0.753	0.99 ± 0.03	0.98 ± 0.03	0.451
**VO_2_peak/BW**	24.1 ± 4.26	26.9 ± 2.32	**0.048**	26.0 ± 4.58	28.9 ± 3.78	0.130	1.09 ± 0.13	1.09 ± 0.15	0.915
**VO_2_peak/NAT-L.Ex**	134 ± 16.5	125 ± 10.7	0.118	139 ± 17.2	132 ± 15.3	0.359	1.04 ± 0.13	1.07 ± 0.16	0.678
**Performance [W]**	101 ± 20.2	144 ± 19.9	**<0.001**	113 ± 13.7	150 ± 18.5	**<0.001**	1.15 ± 0.19	1.05 ± 0.07	0.291
**Performance/NAT-L.Ex**	6.75 ± 1.27	6.65 ± 1.01	0.868	7.12 ± 0.87	6.90 ± 1.12	0.637	1.11 ± 0.18	1.04 ± 0.07	0.189

BMI: body mass index; TAT: total adipose tissue; SCAT: subcutaneous adipose tissue; VAT: visceral adipose tissue; AT: adipose tissue; L.Ex: lower extremities; NAT: non-adipose tissue; IAT: individual anaerobic threshold; BW: body weight; BP: blood pressure: fast.: fasted; ISI_MATS_: insulin sensitivity index estimated by the method of Matsuda and DeFronzo [28]; HbA1C: hemoglobin A1C; VO2peak: peak oxygen consumption; data is presented as mean ± SD; Statistical significance was determined by *t*-test or Wilcoxon for non-normal distributed data, *p* < 0.05, *n* = 24–25 (16f/8-9 m).

**Table 2:** Sex hormone concentrations in serum.

	Baseline			Trained			Fold change		
	Female	Male	*p*-Value	Female	Male	*p*-Value	Female	Male	*p*-Value
**Estradiol [pmol/L]**	342 ± 206	162 ± 23.7	**<0.001**	393 ± 217	202 ± 76.2	**0.022**	1.37 ± 0.84	1.24 ± 0.43	0.771
**Progesterone [nmol/L]**	3.46 ± 3.67	1.81 ± 0.72	0.479	7.02 ± 9.63	1.82 ± 0.54	0.531	5.21 ± 9.57	1.06 ± 0.22	0.766
**Testosterone [nmol/L]**	1.56 ± 0.50	13.3 ± 4.04	**<0.001**	1.54 ± 0.66	14.2 ± 5.07	**<0.001**	0.99 ± 0.31	1.07 ± 0.29	0.544

Data is presented as mean ± SD; Statistical significance was determined by t-test or Wilcoxon for non-normal distributed data, *p* < 0.05, *n* = 23–25 (15-16f/8-9 m).

### 2.3 Biopsy collection

Skeletal muscle biopsies were obtained from the lateral part of vastus lateralis muscle. After local anesthesia, skin, fat tissue, fascia, and the muscle epimysium were cut under sterile conditions using a scalpel, and a piece of muscle was removed using a Bergström needle (Pelomi Medical, Albertslund, Denmark) with suction. Biopsies were taken 8 days before (baseline, B) and 5 days after (trained, T) the 8-week intervention in a resting state 60 min after the end of an OGTT as well as 60 min after the first ergometer exercise bout (acute, A) (Figure S 1). 45 min before the acute exercise bout, participants received a defined breakfast to account for the glucose-induced hormonal changes after the OGTT in the rested state biopsies. All biopsies were collected at 11:00 am ± 30 min.

### 2.4 Proteomics sample processing

Frozen biopsies were lysed in 200 μl pre-cooled sodium deoxycholate (SDC) lysis buffer containing 4% (w/v) SDC and 100 mM Tris–HCl (pH 8.5) in tubes filled with 1.4 mm ceramic beads (0.5 mL CK14 soft tissue homogenizing, Bertin, Montigny-le-Bretonneux, France). The samples were homogenized by shaking twice for 20 s at 5500 cycles per minute separated by a 5 s break in a Precellys 24 homogenizer (Bertin, Montigny-le-Bretonneux, France). After cell disruption, the mixture was boiled for 5 min at 95 °C. Following snap-freezing on dry ice, samples were sonicated for 5 intervals with 1 s pulses, 5 s off and an intensity of 80 (Probe Sonicator EppiShear, ActiveMotif, Carlsbad, USA). A BCA assay (Thermo Fisher Scientific, Waltham, MA, USA) was performed and 800 μg total protein per sample were used as starting material. Reduction and alkylation were performed in a one pot reaction at 45 °C for 10 min using 20 mM chloracetamide (Sigma Alrich, Steinheim, Germany) and 5 mM tris(2-carboxymethyl)phosphine (Bond-BreakerTM TCEP, Thermo Fisher Scientific). In-solution digestion was performed overnight in a ThermoMixer at 2000 rpm at 37 °C with a protein-to enzyme ratio of 1–100 for both LysC (Wako, Osaka, Japan) and Trypsin (Promega, Madison, WI, USA). Tryptic peptides were acidified with 1% trifluoroacetic acid (TFA). 40 μg of the peptide solution was cleared by centrifugation and loaded onto activated (washed first with 100% acetonitrile followed by 30% methanol in 1% TFA and finally 0.2% TFA) three-layer styrene divinylbenzene-reversed phase sulfonated STAGE tips (SDB-RPS 3 M Empore, CDS Analytical, Oxford, PA, USA) as described [30] with minor adjustments: The STAGE tips containing peptides were first washed with 100 μl 1% TFA in ethyl acetate, 100 μl 1% TFA in isopropanol and 150 μl 0.2% TFA. The peptides were eluted with 5% NH4OH in 80% acetonitrile. Samples were dried completely in a SpeedVac (Concentrator plus, Eppendorf, Hamburg, Germany) for 40 min at 45 °C and stored at −20 °C until MS measurement.

### 2.5 Mass spectrometric (MS) measurements

LC-MSMS analysis was performed in data-dependent acquisition (DDA) mode. MS data were acquired on a Q-Exactive HF-X mass spectrometer (Thermo Fisher Scientific) online coupled to a nano-RSLC (Ultimate 3000 RSLC; Dionex). Tryptic peptides were automatically loaded onto a C18 trap column (300 μm inner diameter (ID) × 5 mm, Acclaim PepMap100C18, 5 μm, 100 Å, Thermo Fischer Scientific) at flow rate of 30 μl/min. Peptides were further separated on a C18 reversed phase analytical column (nanoEase MZ HSS T3 Column, 100 Å, 1.8 μm, 75 μm × 250 mm, Waters, Milford, MA, USA) at 250 nl/min flow rate in a 95-minutes non-linear acetonitrile gradient from 3% to 40% in 0.1% formic acid. The high-resolution (60000 full width at half-maximum) MS spectrum was acquired with a mass range from 300 to 1500 *m*/*z* with automatic gain control target set to 3 × 10^6^ and a maximum of 30 ms injection time. From the MS prescan, the 15 most abundant peptide ions were selected for fragmentation (MSMS) if at least doubly charged, with a dynamic exclusion of 30 s. MSMS spectra were recorded at 15000 resolution with automatic gain control target set to 1 × 105 and a maximum of 50 ms injection time. The normalized collision energy was 28, and the spectra were recorded in profile mode.

### 2.6 Proteome data processing – protein identification and label-free quantification

Proteome Discoverer (PD) software (Thermo Fisher Scientific, version 2.4.1.15) was used for peptide and protein identification via a database search (Sequest HT search engine) against the Swissprot human data base (Release 2020_02, 20349 sequences in PD), considering full tryptic specificity, allowing for up to two missed tryptic cleavage sites, precursor mass tolerance 10 ppm, fragment mass tolerance 0.02 Da. Carbamidomethylation of Cys was set as a static modification. Dynamic modifications included deamidation of Asn or Gln, oxidation of Met, and a combination of Met loss with acetylation on the protein N-terminus. Percolator was used to validate peptide spectrum matches and peptides, accepting only the top-scoring hit for each spectrum (high confidence), FDR cutoff values of FDR <1%, and a posterior error probability of <0.01. The final list of proteins complied with the strict parsimony principle.

Protein abundances for quantification were based on peak intensity values of proteotypic peptides. Normalization was performed on total peptide amount to account for sample loading errors. The protein abundances were calculated summing up the single abundance values for admissible peptides. Only proteins quantified in at least 90% of the samples from at least one experimental group were included in the statistical analysis. Missing values (2607, 1.43 %) were imputed using a downshift of 1.5 from the mean and a width of 0.5 from the SD.

### 2.7 Transcriptomic analysis

Total RNA isolation from snap-frozen biopsies and microarray analysis was described in [31]. Tissues were homogenized using a TissueLyser II (Qiagen) and RNA was isolated with the miRNeasy Kit (Qiagen) including DNAse digestion. Only high-quality RNA (RNA integrity number >7, Agilent 2100 Bioanalyzer) was used for microarray analysis. Total RNA was amplified using the WT PLUS Reagent Kit (Thermo Fisher Scientific Inc., Waltham, USA). Amplified cDNA was hybridized on Human Clariom S arrays (Thermo Fisher Scientific). Staining and scanning (GeneChip Scanner 3000 7G) was done according to manufacturer’s instructions. Transcriptome Analysis Console (TAC; version 4.0.0.25; Thermo Fisher)) was used for quality control and to obtain annotated normalized SST-RMA gene-level data. Statistical analyses were performed with R3.6.3/Rstudio [32].

### 2.8 DNA methylation analysis

Genomic DNA was isolated from snap-frozen biopsies at baseline using the Invisorb Genomic DNA Kit II (STRATEC Molecular GmbH, Berlin, Germany). The bisulfite treatment and hybridization of the DNA samples were carried out by Life & Brain (Bonn, Germany). DNA methylation levels were determined by the Infinium® MethylationEPIC BeadChip version 1. The data were processed using R (v.4) packages “meffil” (v.1.3.1) and “ChAMP” (v. 2.24.0) as described earlier [29,33]. Differentially methylated region (DMR) analysis was performed with the champ.DMR() function which utilised the bumhunter algorithm with default settings, 1000 re-samplings, adapted for HG38 reference.

### 2.9 Cell culture

Primary human myoblasts were obtained from the muscle biopsies as described previously [34]. Only baseline biopsies were used. Myoblast isolation and enrichment of CD56+ myoblasts by magnetic bead cell sorting are described in [35]. In brief, satellite cells were released by collagenase digestion and seeded on 15-cm dishes coated with GelTrex™ (Thermo Fisher Scientific, Germany). After two rounds of proliferation in cloning medium (α-MEM:Ham’s F-12 (1:1), 20% (v/v) FBS, 1% (v/v) chicken extract, 2 mM l-glutamine, 100 units/ml penicillin, 100 μg/ml streptomycin, 0.5 μg/ml amphotericin B), CD56-positive myoblasts were enriched (>90%) using MACS microbeads and LS columns (Milteny Biotech, Germany), according to the manufacturer’s protocol, with a 30-min incubation. They were then stored in the gaseous phase of liquid nitrogen. Cell culture surfaces were prepared with a non-gelling thin-layer GelTrex™ coating. Myoblasts (passage 3 after isolation, passage 1 after enrichment) were proliferated in cloning media until 90% confluency. Myotube differentiation was induced on day 0 as described previously [36] and maintained for 7 days in fusion media (α-MEM, 2 mM l-glutamine, 50 μM palmitate, 50 μM oleate (complexed to BSA with a final BSA concentration of 1.6 mg/ml in medium), 100 μM carnitine) with supplementation of 25 ng/ml (13.16 nmol/l) IGF1 (human recombinant IGF1, I3769, Sigma–Aldrich, Germany). During differentiation, myotubes were cultured in the absence or presence of 20 nM, 100 nM or 200 nM testosterone, 1 nM, 5 nM or 10 nM β-estradiol, 20 nM, 100 nM or 200 nM progesterone (Sigma–Aldrich, Germany) or solvent controls (0.012% ethanol). As there was no difference found between non-treated controls and solvent controls, only non-treated controls are depicted in the figures. Medium was changed three times per week, with or without additional hormones and controls, and 48 h before harvest. Mycoplasma-free culture conditions and cells are subjected to regular monitoring and analysis using the MycoAlert Mycoplasma Detection Kit (Lonza, Switzerland).

### 2.10 Quantitative PCR

RNA was extracted employing the RNeasy kit (Qiagen, Germany). For mRNA detection, reverse transcription was carried out with the Transcriptor First Strand Synthesis Kit (Roche, Switzerland). Expression was measured using QuantiFast SYBR Green PCR Mix and QuantiTect Primer Assays (*ATRNL1*: QT00077147; *CHIC1*: QT01151206; *DDX3X*: QT00053928; *DDX3Y*: QT01011465; *EIF1AY*: QT01674575; *EIF1AX*: QT00233492; *ENO3*: QT01666434; *GGT7*: QT00058002; *GPD1*: QT00098322; *GRB10*: QT00031276; *IRX3*: QT00227934; *KDM5C*: QT01666931; *LDHA*: QT00001687; *LDHB*: QT00071512; *MYBPC1*: QT00078407; *MYBPC2*: QT00014882; *MYBPH*: QT00000588; *MYH1*: QT01671005; *MYH2*: QT00082495; *MYH3*: QT00068439; *MYH4*: QT01668779; *MYH6*: QT00030807; *MYH7*: QT00000602; *MYL3*: QT00090223; *PGK1*: QT00013776; *PHKA1*: QT00040642; *PLIN2*: QT00001911; *RPS28*: QT02310203; *RPS4Y1*: QT01670613; *STS*: QT00084161; *TAF9B*: QT01666280; *TBP*: QT00000721; Qiagen, Germany) in a LightCycler 480 (Roche, Switzerland). Standards were generated by purifying the PCR-product (MinE-lute PCR Purification Kit, Qiagen, Germany) and 10-fold serial dilution. Relative quantification was performed by normalizing expression to the mean of housekeeping genes RPS28 and TBP.

### 2.11 Statistical analyses

Linear regression models were analyzed with R4.1.1/RStudio [32]. Normality was tested by Shapiro-Wilk-test from the R package ‘stats’ (v3.6.3) and non-normal data were log-transformed. Differences between groups or time points were calculated using (paired) limma analysis. All linear models included age as a covariate to account for the slight age imbalance between sexes. Differential analyses were performed using limma with empirical Bayes moderation to accommodate unequal group sizes. For time points comparisons the fold change was calculated. In the case of *p*-value adjustment a linear model was calculated (Benjamini-Hochberg post hoc adjustment). Differences between individual groups were assessed using one-way ANOVA with Fisher’s LSD post hoc test or Bonferroni correction for multiple comparisons when appropriate. Graphs were made using the R packages ‘ggplot2’ (v3.3.2) and ‘ggrepel’ (v0.9.1) and figures assembled using InkScape (v1.0). Functional enrichment analysis was carried out online using https://biit.cs.ut.ee/gprofiler/with *p* < 0.05 as threshold and g:SCS as method for multiple testing correction. Homo sapiens was chosen as organism and analysis was performed using the databases from gene ontology biological process (GO:BP), cellular components (GO:CC), molecular functions (GO:MF), Reactome (REAC), KEGG, Wikipathways (WP) and human protein atlas (HPA). MitoCarta 3.0 was used for the annotation to mitochondria.

## 3 Results

### 3.1 Study cohort characteristics

The study cohort, training regime, and inclusion criteria have been described elsewhere [25]. As the initial study design was not focused on sex-specific differences our study cohort included more females than males (Table 1). The age range of the subjects was similar for both females and males, with a slight statistical difference (females: 28 years, males: 33 years). Statistical analysis was adjusted for age. By study design, all participants had a BMI >27 (11 participants >30) with no difference between sexes (Table 1). All had a sedentary lifestyle (<120 min of physical activity per week). At baseline, females had higher total adipose tissue, subcutaneous adipose tissue, and adipose tissue content in lower extremities, while males had higher visceral adipose tissue and non-adipose tissue content in the lower extremities. Males had higher VO_2_peak/body weight and performance in Watt [W] when related to body weight, but not after correction to leg lean mass. The 8-week training intervention improved aerobic capacity and reduced adipose tissue similarly in both sexes. The only statistically significant differences were found in fold changes in lower extremity tissues, with a significant increase in non-adipose tissue in females.

### 3.2 Pronounced differences on multi-OMICs level between female and male skeletal muscle at baseline

**Figure 1:**
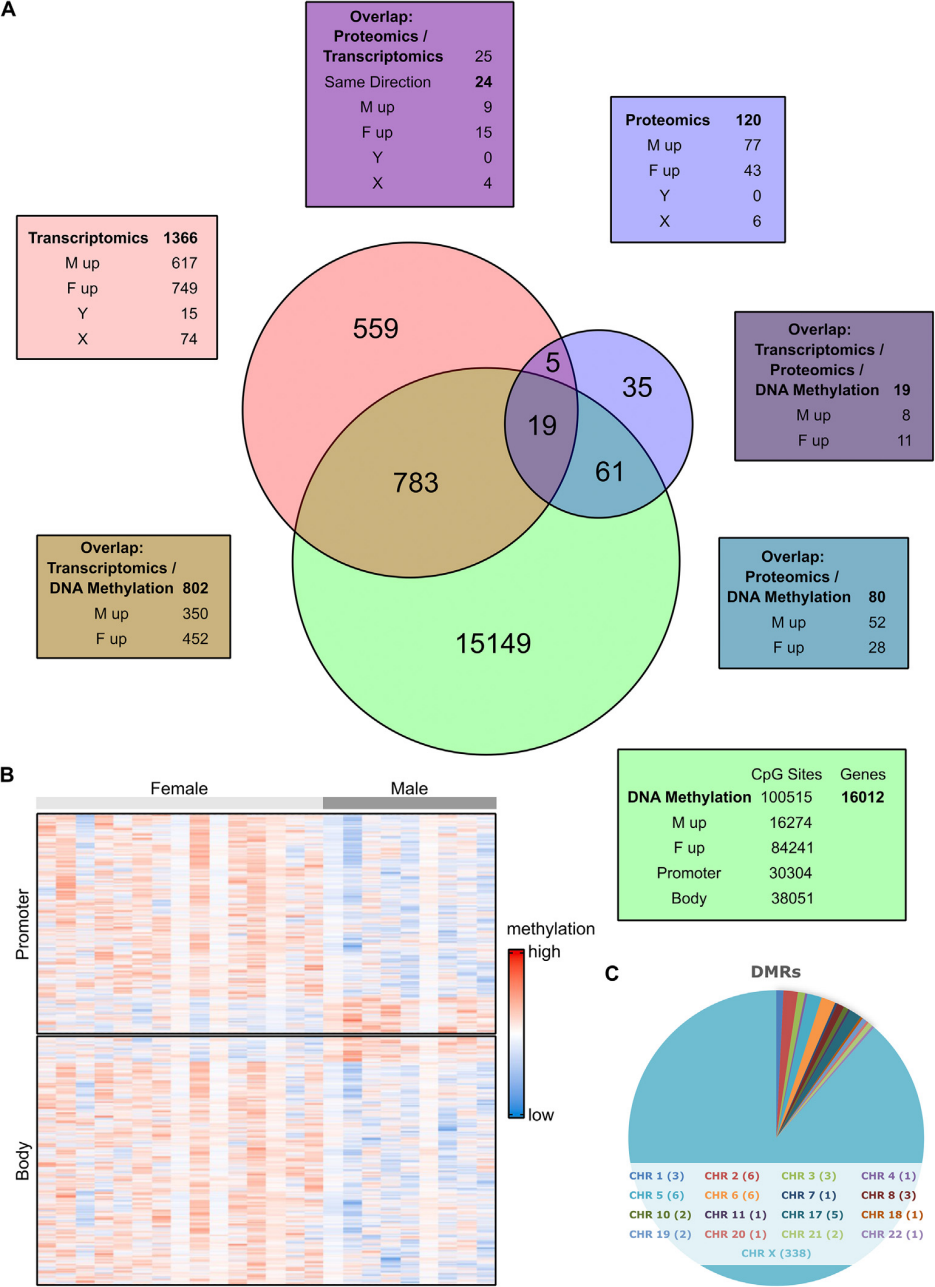
Multi-Omics analysis of female vs. male skeletal muscle at baseline. A) Skeletal muscle biopsies obtained at baseline were analyzed for transcriptomic, proteomic and epigenomic (DNA-methylation) differences between females (F) and males (M). Numbers show the sex differences found in the datasets as indicated. Venn diagram visualizing the overlap of the differences in the multi-omics datasets. B) Heatmap depicting scaled methylation levels of 2,192 CpG sites located in 802 differentially expressed genes. Each column represents a skeletal muscle sample from an individual donor, each row indicates the methylation level of a single CpG site with significant differences between males and females. Rows marked in light grey represent female participants, male participants are indicated in dark grey. The differentially methylated CpG sites found in the promoters are displayed in the upper part of the heatmap, those located in gene bodies are shown in the lower part. C) Pie chart of identified differentially methylated regions (DMRs) between females and males. Statistical significance was determined by limma t-test Bayes *p* < 0.05, *n* = 25 (16f/9 m).

We analyzed the skeletal muscle transcriptome, proteome and methylome data obtained from the skeletal muscle biopsies collected in the resting state before the 8-weeks endurance exercise intervention for sex differences. Not considering sex-specific differences, the methylome and transcriptome data from this cohort were reported recently [29]. We now identified 100,515 differentially methylated CpG sites in 16,012 genes (*p* < 0.05) and 1,366 differentially abundant transcripts (*p* < 0.05) in female and male skeletal muscle (Figure 1A, Supplementary Data Table 1). The proteome data set comprised 1,857 proteins identified with high confidence and quantified in at least 90% of all samples at one time point. We identified 120 differentially abundant proteins (*p* < 0.05) between female and male skeletal muscle at baseline (Figure 1A, Supplementary Data Table 1). Of all differentially methylated CpG sites, 84,240 (83.8%) were hypermethylated in females. DNA methylation in promoter/enhancer regions is associated with gene silencing, high DNA methylation in the gene-body with active transcription [37]. Considering this, differential expression of 802 (58.7%) transcripts could be linked to changes in DNA methylation of 2907 CpG sites. The high proportion of elevated methylation levels in female participants was similarly detected in the promoters and gene bodies of the corresponding 802 genes (Figure 1B, Supplementary Data Table 1). Sex-specific abundance of 80 (66.6%) proteins could be linked to DNA methylation, and a total of 19 differentially abundant proteins could be traced back over differential gene expression to sex-specific DNA methylation (Figure 1A, Supplementary Data Table 1). To further investigate genomic regions with consistent and extended differential methylation, we calculated differentially methylated region (DMR). The total number of DMRs was 382 of which 375 were in close proximity to 452 genes and 24 in intergenic regions. The majority of the DMRs (88%) were located in ChrX (Figure 1C, Supplementary Data Table 1). These findings highlight deeply rooted distinctions in the epigenome of untrained, overweight/obese males and females which could potentially explain up to 60% of transcriptomic and proteomic sex differences.

### 3.3 Transcriptomic differences at baseline underline an influence of sex on glucose and lipid metabolism in skeletal muscle

**Figure 2:**
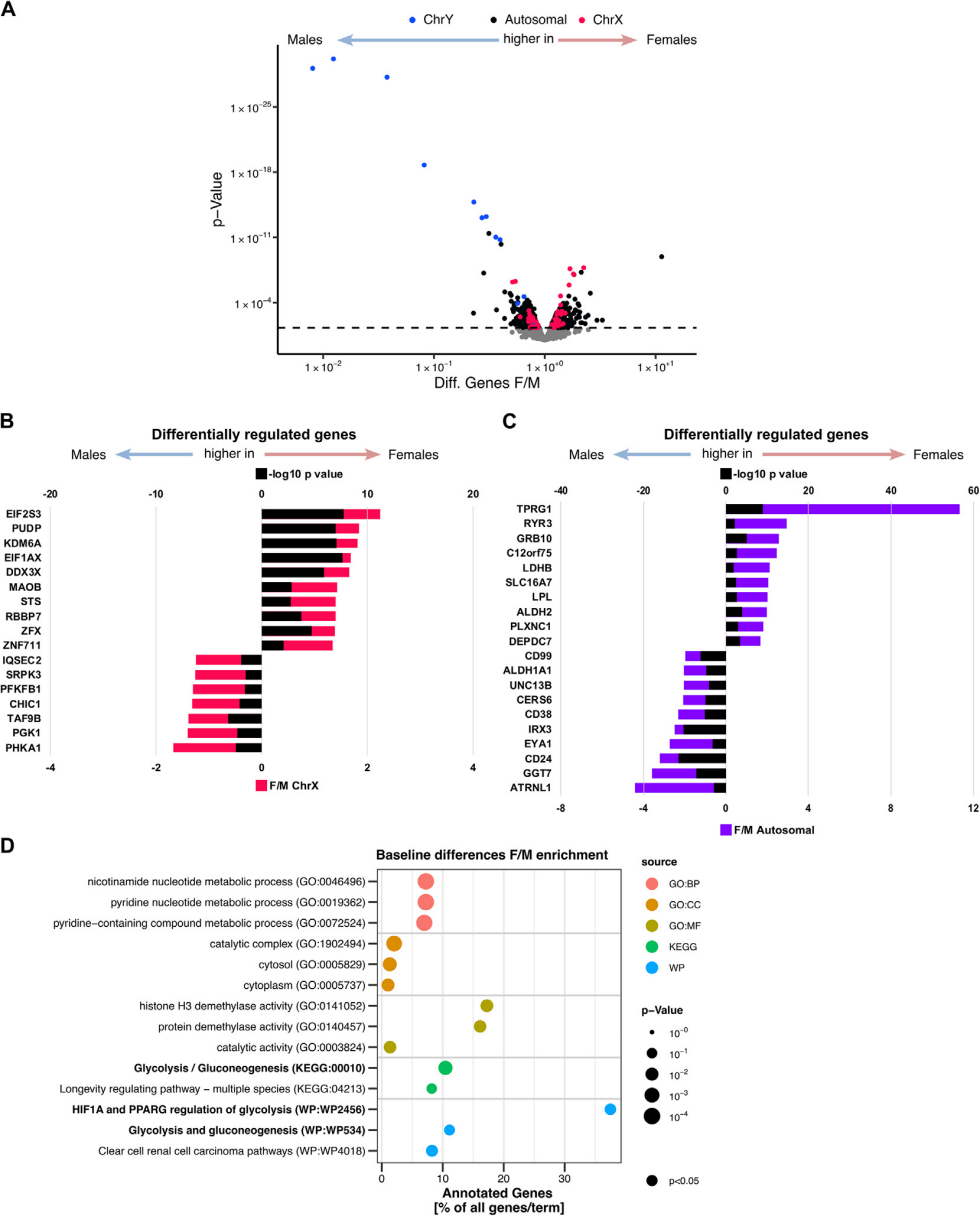
Transcriptomic analysis of female vs. male skeletal muscle at baseline. Skeletal muscle biopsies obtained at baseline were analyzed for transcriptomic differences between females and males. A) Volcano plot of differentially expressed transcripts being higher in males (left) or higher in females (right), X-chromosomal genes are labeled in red, Y-chromosomal genes are labeled in blue. Top 10 transcripts higher expressed in males (left) and females (right) conserved in this and another independent cohort [38] are plotted, fold change (F/M) in B) X-chromosomal (red), C) autosomal (purple) genes (bottom axis), -log10 *p*-values are plotted in black (top axis). D) Enrichment analysis based on the 196 differentially expressed genes in both cohorts. Statistical significance was determined by limma t-test Bayes *p* < 0.05, *n* = 25 (16f/9 m) and validation cohort (*n* = 19; 12f/6 m).

In the transcriptome, the most pronounced differences were found in sex chromosomal transcripts dominated by ChrY-located transcripts in males (Figure 2A, Figure S2A). In contrast, higher expression of ChrX-located transcripts was evenly distributed among sexes. The majority of transcriptomic differences was found in autosomal genes (Figure 1, Figure 2A). The transcriptomic differences were validated by comparison with the skeletal muscle transcriptome data of a previously published independent second cohort consisting also of sedentary subjects with overweight/obesity participating in an exercise intervention study [38], yielding 196 conserved transcripts (Supplementary Data Table 1). Key metabolic genes differed: *GRB10*, *LDHB*, and *LPL* were higher expressed in females, while *ALDH1A1*, *PFKFB1*, *PGK1*, and *PHKA1* were higher in males (Figure 2B,C). Pathway enrichment pointed to sex-specific differences in glycolysis and demethylase activities (Figure 2D). Next, we examined the impact of the differentially methylated regions on gene expression. In total, seven DMRs were linked to transcripts exhibiting sex specific differential expression (Figure S2B). Except of one, all the DMRs were located in chromosome X and more specifically in promoter regions of the corresponding genes. These seven DMRs displayed female specific hypermethylation, which correlated with reduced expression levels of *PGK1*, *PHKA1*, *PFKB1*, *CHIC1*, *SRPK3*, *IQSEC2* and *GTDC1* (Figure S2B,C, Supplementary Data Table 1). Thus, the higher expression of key enzymes of glycogen degradation and glycolysis in male muscle is linked to sex-specific DMRs.

### 3.4 Proteomic differences provide a molecular basis for sex-specificity of glucose and lipid utilization and fiber type prevalence

**Figure 3:**
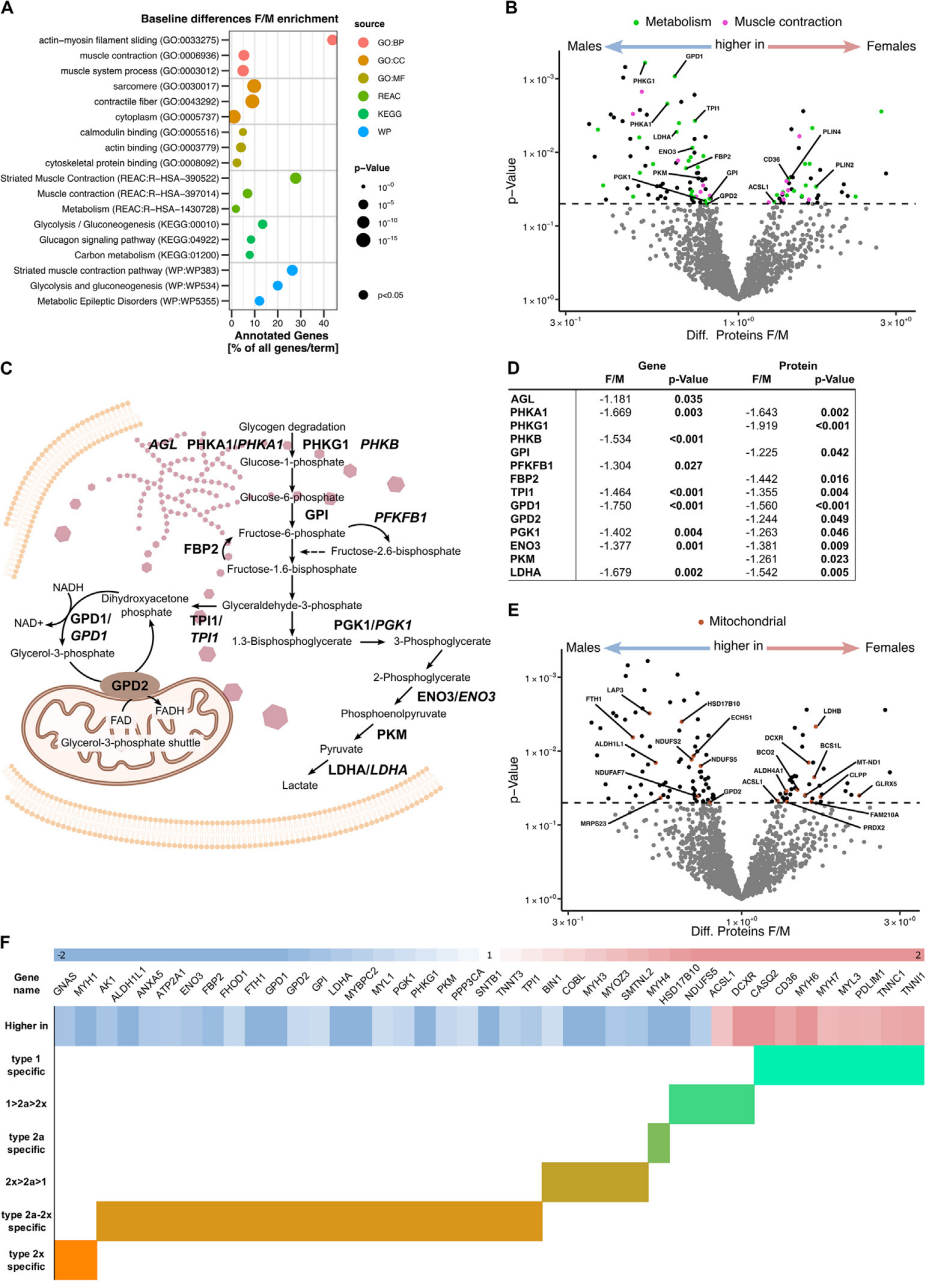
Proteomic analysis of female vs. male skeletal muscle at baseline. Skeletal muscle biopsies obtained at baseline were analyzed for proteomic differences between females and males. A) Enrichment analysis based on the 120 differentially abundant proteins between females and males. B) Volcano plot of differentially abundant proteins being higher in males (left) or higher in females (right). Color-coded for proteins enriched in the top terms (REAC database) Metabolism (green) and Muscle contraction (pink). Key enzymes of glycogen, glucose and lipid metabolism are labeled. C) Schematic representation of the glycogen degradation and glycolysis pathway and the key enzymes (PROTEIN/*GENE*) found to be elevated in male skeletal muscle. D) Table of differentially expressed genes and proteins featured in C). E) Volcano plot of differentially abundant proteins being higher in males (left) or higher in females (right) as shown in (A), proteins with mitochondrial localization are labeled. F) Heatmap of proteins with differential abundance between females and males (red higher in females, blue higher in males), with higher percentage of abundance in oxidative slow type 1 fibers and fast type 2a and 2x fibers based on [39]. Statistical significance was determined by limma t-test Bayes *p* < 0.05, *n* = 25 (16f/9 m).

In the skeletal muscle proteome, enrichment analysis of the 120 proteins differentially abundant between females and males indicated sex-specific expression of actin-myosin filament proteins and enzymes of glucose metabolism (Figure 3A). Among these are many key enzymes of glycogen degradation and glycolysis, and differential expression was also found on transcriptomic level (Figure 3B–D). Lipid metabolism-related proteins (ACSL1, CD36, PLIN2, PLIN4) were higher in females (Figure 3B). Mitochondrial proteins, based on MitoCarta 3.0, were evenly distributed between sexes (Figure 3E). Elevated glucose/glycogen utilization in male muscle can be associated with the fast-twitch 2 A and 2X muscle fibers. Thus, we overlapped our proteomic results with the list of human fiber type-specific proteins reported by Murgia et al., in 2021 [39] (Figure 3F). Proteins specific to fast-twitch glycolytic fibers were more abundant in males, those associated with slow-twitch oxidative fibers more abundant in females (Figure 3F). This proteomic profile aligns with higher fast-twitch fiber prevalence and carbohydrate reliance in males, while female exhibit proteomic profiles suggesting more slow-twitch fibers and high lipid utilization. Of the proteins with fiber type-specific preference (Figure 3F) only proteins with metabolic function (ENO3, GPD1, LDHA, PGK1, TPI, ACSL1, CD36) and calsequestrin CASQ2 were also different at transcript level (Supplementary Data Table 1). The sex-specific abundance of proteins representing the type 1 and 2 fiber-specific contractile and structural profile appears to be post-transcriptionally regulated.

### 3.5 Sex influences the transcriptional response to acute exercise

**Figure 4:**
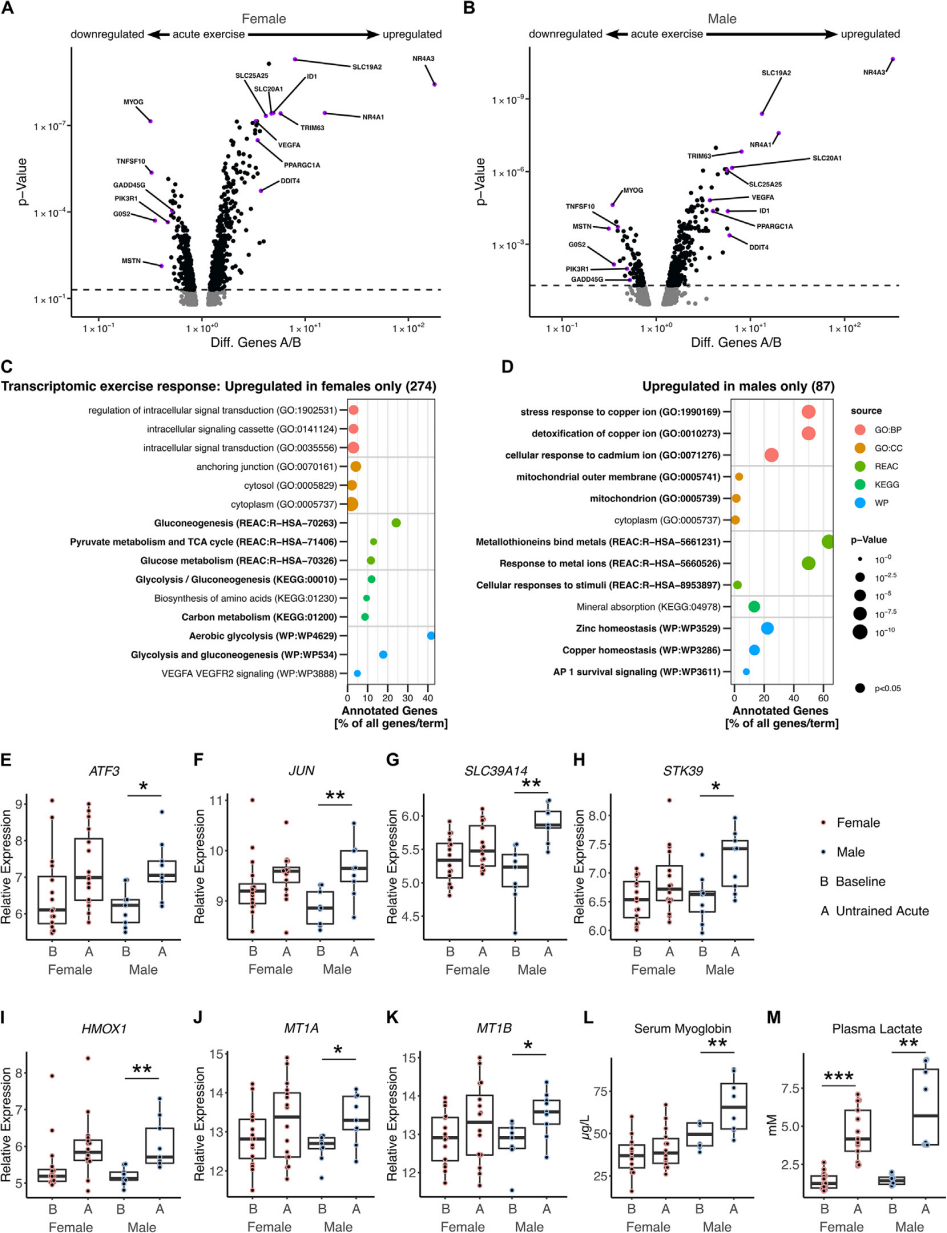
Differences in the transcriptional response of female and male skeletal muscle to acute exercise. A, B) Volcano plots of differentially regulated transcripts in females and males after the first acute bout of exercise compared to baseline (A/B). Enrichment analysis based on C) 274 transcripts upregulated in female skeletal muscle only and D) 87 transcripts upregulated in male skeletal muscle only in response to acute exercise. Relative transcript abundance of E) *ATF3*, F) *JUN*, G) *SLC39A14*, H) *STK39*, I) *HMOX1*, J) *MT1A* and K) *MT1B* in skeletal muscle of females and males at baseline (B) and 60min after the first acute exercise bout (A). Statistical significance was determined by limma t-test Bayes BH, *p* < 0.05, *n* = 25 (16f/9 m), ∗*p* < 0.05, ∗∗*p* < 0.01. Serum myoglobin L) and plasma lactate M) was measured in females and males at baseline (B) and 5min after the first acute exercise bout (A). Statistical significance was determined by one-way ANOVA LSD, *n* = 22 (14f/8 m), ∗∗*p* < 0.01.

Next, we studied whether the initial response of skeletal muscle to acute exercise differs between sexes as well. We focused on the transcriptomic response since the skeletal muscle biopsies were collected 60 min after the first 30 min-ergometer exercise bout, when particularly a transcriptional regulation response was observed (Figure S3A). The regulation of transcripts that have a strong response to acute exercise (e.g. upregulation of *NR4A3*, *NR4A1*, *PPARGC1A*, *SLC19A2*, *VEGFA* and downregulation of *G0S2*, *MYOG*, *MSTN*) [40] was highly comparable between sexes after the first acute exercise bout (Figure 4A,B). In addition, females upregulated 274 transcripts which were not regulated in males. The transcripts were enriched in pathways of glucose metabolism, glycolysis, pyruvate metabolism and TCA cycle (Figure 4C). Male skeletal muscle showed a distinct transcriptomic response. The upregulated 87 transcripts, which were not regulated in females, were enriched in mitochondrial and oxidative stress-related pathways (Figure 4D). This includes pronounced upregulation of cellular stress-inducible transcription factors *ATF3* and *JUN,* protein kinase *STK39*, the divalent metal transporter *SLC39A14*, and oxidative stress-responsive *HMOX*, *MT1A* and *MT1B* transcripts (Figure 4E–K). Serum myoglobin concentrations serve as marker of increased myofiber damage and skeletal muscle membrane vulnerability. In line with elevated transcriptional markers of cellular stress in male skeletal muscle, serum myoglobin levels were increased after the acute exercise bout only in males (Figure 4L). Baseline myoglobin values were also by trend higher in males than in females (*p* = 0.08), presumably due to the higher muscle mass of males. Plasma lactate was increased to a similar concentration in both sexes (Figure 4M).

The acute exercise bouts were performed at an individual intensity corresponding to 80% of VO_2_peak. Accordingly, heart frequency per minute (159 ± 12 vs. 150 ± 17, *p* = 0.188) and rating of perceived exertion of leg work (15 vs. 16, *p* = 0.518, corresponding to BORG scala) during the first 30 min-ergometer exercise bout were not different between sexes, despite the lower absolute intensity in females (101 ± 20 vs. 143 ± 20 W, *p* < 0.001). Of note, the absolute intensity of the performed exercise bout did not correlate with the upregulation of *ATF3*, *JUN*, *STK39*, *SLC39A14*, *HMOX*, *MT1A* and *MT1B* transcripts (Supplementary Table 1).

### 3.6 Eight weeks of endurance training increase key enzymes of mitochondrial metabolism in both sexes and equalize baseline differences

**Figure 5:**
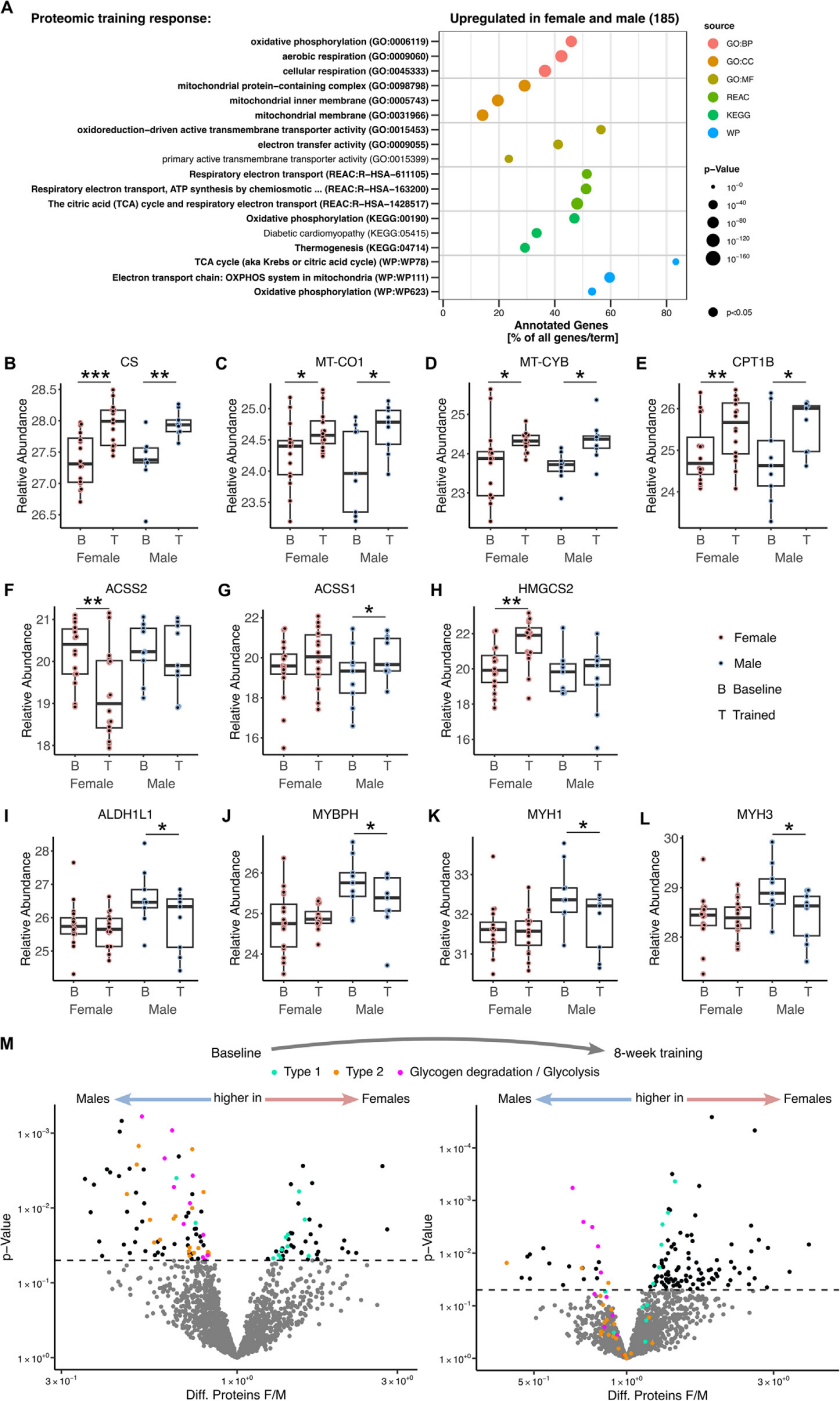
Effects of 8-week training on skeletal muscle proteome of females and males. A) Enrichment analysis based on 185 proteins upregulated in both female and male skeletal muscle in response to 8 weeks of training. Relative protein abundance of B) CS, C) MT–CO1, D) MT-CYB, E) CPT1B, F) ACSS2, G) ACSS1, H) HMGCS2, I) ALDH1L1, J) MYBPH, K) MYH1 and L) MYH3 in skeletal muscle of females and males at baseline (B) and after 8 weeks of training (T). K) Volcano plots of differentially abundant proteins at baseline and after 8 weeks of training, being higher in males (left) or higher in females (right), color-coded for differentially abundant proteins at baseline, turquoise for oxidative slow type 1 fibers and orange for fast type 2 fibers based on (Murgia et al., 2021). Proteins relevant for glycogen degradation/glycolysis were shown as pink dots. Statistical significance was determined by limma t-test Bayes, *p* < 0.05, *n* = 25 (16f/9 m), ∗*p* < 0.05, ∗∗*p* < 0.01, ∗∗∗*p* < 0.001.

To elucidate the sex-specific response to the 8-week endurance training intervention, we focused on the proteomic response. As the skeletal muscle biopsies were obtained 5 days after the final training session, mainly sustained changes in the proteome, but fewer changes in the transcriptome were detected (Figure S3A). At first view, the proteomic response was highly comparable in both sexes. Both females and males upregulated 185 proteins enriched in oxidative phosphorylation, mitochondrial respiration and ATP synthesis (Figure 5A). Common protein responses to the 8-weeks training included upregulation of mitochondrial marker proteins and key enzymes of the TCA cycle, respiratory chain and β-oxidation as exemplarily shown for CS, MT–CO1, MT-CYB and CPT1B (Figure 5B–E). The mitochondrial proteomic response aligns with our previous findings from this cohort, in which isolated myofibers exhibited significantly increased mitochondrial respiration after training similarly in females and males [25]. This supports the functional relevance of the observed proteomic adaptations.

Among sex-specific responses were alterations in key enzymes of acetyl-CoA metabolism. Cytosolic acyl-CoA synthetase short chain ACSS2, which is involved in lipid synthesis, was reduced only in female muscle, whereas the mitochondrial ACSS1 was increased only in male muscle after 8 weeks of training (Figure 5F,G). The key enzyme of ketogenesis HMGCS2 was upregulated only in female muscle (Figure 5H). Among the proteins found to be reduced only in male muscle in response to the 8-weeks training were the fast-twitch and glycolytic fiber type-specific proteins ALDH1L1, MYBPH, MYH1 and MYH3, resulting in equalized protein levels between sexes after training (Figure 5I–L). Based on this observation, we reevaluated all fiber type-specific proteins that were initially different between females and males at baseline (Figure 3D) and found that most of those were no longer differentially abundant after 8 weeks of endurance exercise (Figure 5M). Similarly, the abundance of enzymes involved in glycogen degradation and glycolysis that were initially higher in males at baseline (Figure 3B–D) was reduced in male muscle toward the levels of females (Figure 5M). In summary, 8 weeks of controlled endurance exercise equalized initially observed differences in skeletal muscle toward a common metabolically beneficial response in females and males.

### 3.7 Hormonal regulation of sex-specific differences observed *in vivo* in myotubes *in vitro*

Lastly, we examined whether sex-specific differences found *in vivo* persisted *in vitro* by investigating fully differentiated myotubes cultivated from satellite cells of the baseline biopsies (Figure S4A). X-chromosomal genes *EIF1AX*, *DDX3X*, *STS*, and *KDM5C* retained higher expression in female myotubes while no difference was observed for X-chomosomal *PHKA1*, *PGK1*, *TAF9B* and *CHIC1,* higher in male skeletal muscle *in vivo* (Figure S4B). Autosomal differences were largely absent except for higher *GRB10* (*p* = 0.096) by trend and *TPRG1* expression in female myotubes (Figure S4C, D).

**Figure 6:**
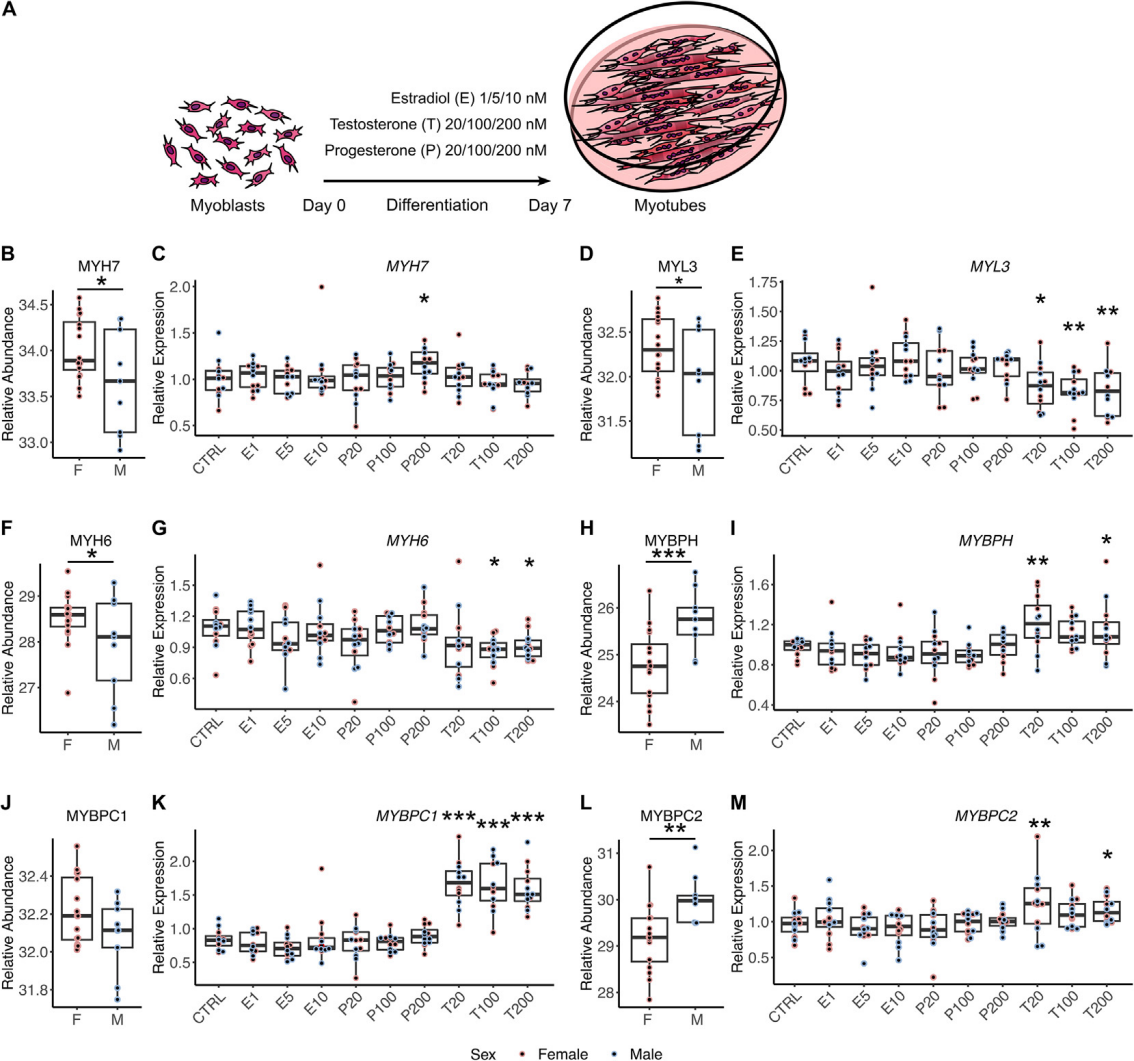
Sex hormone-driven transcriptional regulation of fiber type-specific proteins in myotubes *in vitro*. A) Myoblasts derived from 6 female and 6 male donors were cultured and differentiated into myotubes for 7 days. During differentiation, myotubes were treated with estradiol (E) (1/5/10 nM), progesterone (P) (20/100/200 nM) or testosterone (T) (20/100/200 nM) or left untreated (CTRL). RNA expression of C) *MYH7*, E) *MYL3*, G) *MYH6*, I) *MYBPH*, K) *MYBPC1* and M) *MYBPC2* in myotubes after hormonal treatment, respectively. Differences in RNA expression were determined by one-way ANOVA Bonferroni, *n* = 12 (6f/6 m), ∗*p* < 0.05, ∗∗*p* < 0.01, ∗∗∗*p* < 0.001. To the left is the respective protein abundance in skeletal muscle of females (F) and males (M) at baseline (B, D, F, H, J, L). Statistical significance was determined by limma t-test Bayes *p* < 0.05, *n* = 25 (16f/9 m), ∗*p* < 0.05, ∗∗*p* < 0.01, ∗∗∗*p* < 0.001. Red dots, female donor; blue dots, male donor.

We further investigated whether the differences found *in vivo* but were absent *in vitro*, can be induced by the presence of the sex hormones estradiol, progesterone or testosterone during myotube differentiation. Treatment with sex hormones for 7 days during differentiation, at physiological serum concentrations of either females or males or 5 to 10 times higher, induced some transcriptomic changes (Figure 6A). Slow-twitch marker *MYH7* was slightly induced by 200 nM progesterone on transcriptional level. Testosterone already at a concentration found in serum of males upregulated fast-twitch fiber genes (*MYBPH*, MYBPC2) and downregulated slow-twitch genes (*MYL3*, *MYH6*) (Figure 6B–M). A strong inducing effect of testosterone was also found on *MYBPC1* (Figure 6K). Estradiol reduced *LDHA* expression at 10 nM (Figure S5E). Other *in vivo* differences (*MYHs*, *LDHB*, *IRX3*, *GPD1*, *ENO3*, *PLIN2*) could not be evoked or were regulated in the opposite direction (*MYH2*) (Figure S5). In conclusion, the treatment of human myotubes with sex hormones can to some extent restore the *in vivo* transcriptomic differences.

## 4 Discussion

In this study, we provide an in-depth molecular analysis of differences between female and male skeletal muscle by applying a multi-omics approach employing DNA methylation, transcriptomics and proteomics. We investigated the differences in untrained muscles, but also the impact of sex on the response to acute exercise and to an 8-week endurance training by analyzing three skeletal muscle biopsies per subject obtained before training, after the first exercise bout and 5 days after the final exercise session.

In untrained muscles, our overlap analysis of transcriptome and methylome data revealed that nearly 60% of differentially expressed genes and proteins are potentially mediated by sex-specific DNA methylation patterns, indicating DNA methylation as a key contributor to metabolic differences in skeletal muscle prior to exercise intervention. Consistent with previous findings, we detected overall higher methylation levels in females [22,41]. Landen et al. examined differentially methylated regions (DMRs) in skeletal muscle from 222 males and 147 females, identifying 10,240 DMRs, 94% hypermethylated in females [22]. Davegardh et al. similarly reported hypermethylation of DMRs in female skeletal muscle and myoblasts [41]. This phenomenon extends beyond skeletal muscle, as hypermethylation of autosomal genes is also observed in female leukocytes and umbilical cord blood [42,43]. Our data showed that the hypermethylation is evenly distributed between promoter regions and gene bodies, with a transcriptional regulation that aligns to the differences in DNA methylation especially for hypermethylated DMRs in females corresponding with higher expression of key enzymes of glycogen degradation and glycolysis in male muscle. The extent of hypermethylation, independent of genomic location, implies a global difference in enzymatic methylation or demethylation activity, though the underlying mechanism remains unclear [22].

Sexual dimorphism in skeletal muscle transcriptomes has been investigated in prior studies by either microarray analysis as we did [22,23] or RNA sequencing [21]. A comparison of 196 differentially expressed transcripts in our two exercise cohorts with the most recent RNA sequencing data of young adults (18–30 years) by Pataky et al. revealed 141 overlapping transcripts, including 111 autosomal genes [21]. Genes such as lipoprotein lipase (*LPL*), acyl-CoA synthetase short-chain (*ACSS2*), insulin receptor (*INSR*), growth factor receptor-bound protein (*GRB10)*, the key antioxidant enzyme catalase (*CAT)* and ryanodine receptor type 3 (*RYR3)* showed higher expression in females, supporting the observed increased triglyceride clearance capacity, antioxidant activity, and insulin sensitivity in female skeletal muscle. As ablation of GRB10, a modulator of the proximal IGF1 and insulin signaling, in mice leads to muscle hypertrophy and reduced insulin signaling [44], higher GRB10 expression in females might contribute to smaller muscle mass. Glycolytic enzymes such as *ENO3*, *GPD1*, *LDHA*, and *TPI1* were upregulated in males, reflecting their reliance on glucose metabolism. Highly conserved is the male-specific upregulation of *IRX3* alongside with sex-specific promoter hypomethylation and gene body hypermethylation [[21], [22], [23]]. Elevated IRX3 in mouse skeletal muscle was discussed as potentially androgen-regulated [45], however our data did not show upregulation of *IRX3* transcripts in testosterone-treated myotubes irrespective of donor sex. While the function of IRX3 in skeletal muscle remains unclear, its involvement in adipose tissue regulation suggests potential metabolic significance [46].

Pronounced sexual dimorphism in skeletal muscle was evident on the proteomic level and underpins differences in metabolism, with females favoring lipid metabolism and males exhibiting enhanced glycogen degradation and glycolysis. This aligns with a prior study in non-overweight, untrained individuals, where females exhibited higher protein levels associated with fatty acid degradation and amino acid metabolism, while males showed increased carbohydrate metabolism and proteasome pathway proteins [24]. Differences in the proteome such as PHKA1, PGK1, TPI1, GPD1, LDHA (male-elevated) and PLIN4, CD36, ACSL1 (female-elevated) were reflected by transcriptomic and DNA methylation analyses. This deeply rooted sexual dimorphism in glucose and lipid metabolism correlates with a fiber type-specific proteome and sex-specific fiber type composition, where females have a higher proportion of slow-twitch oxidative type 1 fibers and males possess more fast-twitch type 2 fibers [14,[47], [48], [49], [50]]. More slow-twitch fibers can support endurance exercise and fatigue resistance in females [51], while more fast-twitch fibers provide higher power output and faster contraction rates, contributing to the greater muscle strength and better sprint distance performance in males [52]. Unlike proteome data, our transcriptomic analysis did not highlight fiber type differences, consistent with previous transcriptome studies [21,22]. While the abundance of many fiber type-specific metabolic enzymes and regulators can be traced back to the transcriptional and epigenetic level, this was not observed for contractile apparatus proteins raising the question of post-transcriptional mechanisms responsible for the sexual fiber type dimorphism. Translation initiation factors, ribosomal proteins, and a ribosome maturation factor are among the 120 proteins different at baseline but a specific contribution of these factors to a divergent translation of sex-specific contractile structure proteins needs to be elucidated.

While previous studies mainly examined cumulative responses to long-term training [21,22,53], we also analyzed biopsies collected after a single endurance exercise session. Our findings revealed a clear sex difference in the initial transcriptomic response: females upregulated transcripts associated with aerobic glycolysis, pyruvate metabolism, and the TCA cycle, while males exhibited a stronger response related to cellular and oxidative stress. Well in line, males have higher levels of circulating muscle damage and oxidative stress markers following acute endurance or strength exercise bouts [[54], [55], [56]]. The protective effects of estradiol, which reduces mitochondrial oxidative damage, enhances antioxidant capacity, and potentially protects skeletal muscle membranes preventing leakage of muscle proteins such as myoglobin or creatine kinase into circulation, may explain this disparity [[57], [58], [59]]. One caveat comparing acute individualized exercise responses is that males trained at higher absolute intensities and engaged greater muscle mass. While the differential regulation of the stress-responsive transcripts does not significantly correlate with achieved performance in [W], the effect of active muscle mass on muscle temperature needs to be considered.

The comparison of the proteomic response after 8 weeks of training between females and males does not indicate any harmful consequences of the initial stress response of male muscle. The proteomic adaptation to eight weeks of endurance training was largely similar between sexes. Both males and females upregulated proteins involved in the TCA cycle, oxidative phosphorylation, and β-oxidation, consistent with improved aerobic capacity. Our previous analysis of the increase in mitochondrial respiration by training in isolated myofibers revealed no differences between sexes [25], supporting our proteomic findings. We also did not observe differences in baseline muscle mitochondrial respiration, in contrast to subcutaneous adipose tissue with higher respiration in females. Thus, the higher oxidative fiber content in female muscle is not reflected by higher mitochondrial respiration even if adjusted for fiber type proportion based on the abundance of MYH1, 2 and 7.

Some key enzymes of acetyl-CoA metabolism showed a divergent regulation in females and males after the 8 weeks of training. The reduction of the cytosolic ACSS2 but not the mitochondrial ACSS1 enzyme in female muscle after training may favor reduced lipid synthesis and pave the way for enhanced supply of acetyl-CoA for citrate synthesis and fueling the TCA cycle. HMGCS2 was found to be increased only in female muscle after the 8 weeks training. HMGCS2 catalyzes the second and rate-limiting step of ketogenesis. Ketone production is not considered to play a major role in skeletal muscle but recently, β-hydroxybutyrate was described as protective factor for skeletal muscle by preventing muscle mass loss, mitochondrial impairments and functional decline [60]. The intramuscular production of β-hydroxybutyrate can be a yet underestimated contributor to this mechanism, in addition to local ketone bodies serving as alternative fuel for skeletal muscle during exercise. The regulation of enzymes of acetyl-CoA synthesis and metabolism after 8 weeks of endurance training can be an adaptive process particularly in females, shifting acetyl-CoA from lipid synthesis to mitochondrial TCA cycle and ketogenesis thereby supporting increased fuel oxidation and protective mechanisms.

In general, the proteomic adaptations in both sexes align with increased aerobic endurance performance. In males, a reduction in fast-twitch type 2 fiber-specific proteins and overall reduction of sex-specific differences in glycolytic enzymes suggest a fiber type shift towards oxidative fibers. Both sexes exhibited an upregulation of type 1 fiber-specific proteins. Previous studies showed that endurance-trained individuals have a significantly higher abundance of mitochondrial proteins, oxidative phosphorylation proteins, TCA cycle enzymes, and fatty acid metabolism proteins [24]. This signature is consistent with the characteristics of type 1 (slow-twitch) muscle fibers, and suggests long-term endurance training adaptations, enhancing aerobic capacity and energy efficiency in these muscles. Notably, when comparing endurance-trained males and females, only one protein differed, compared to 30 proteins in untrained groups [24]. Overall, the results of our study and of others hint towards the idea that regular endurance training might, over years, reduce or even eliminate sex-based proteomic differences in skeletal muscle, leading to a more similar proteomic profile. We now show that this harmonization already starts after 8 weeks of endurance training. Whether strength training would result in a similar equalization toward a more glycolytic fiber type remains unclear.

Lastly, in a first attempt to understand the regulation of the sex-specific expression of autosomal genes *in vivo*, we studied the expression in cultured myotubes obtained from the muscle biopsy donors. Most of the differences were not conserved *in vitro*, which is consistent with a previous study [41] and suggests a critical role for systemic factors, such as sex hormones, in shaping muscle metabolism [61]. Our data show that primarily testosterone can drive sex-specific transcriptional regulation in both female and male myotubes, but several sex-dependent transcriptomic differences observed *in vivo* could not replicated by hormonal treatment *in vitro*. Previous studies also demonstrated that myotubes obtained from female and male donors respond similarly to treatment with testosterone or estradiol [21,62]. The obvious sex dimorphism in the expression in histone demethylases located on X and Y chromosomes (*KDM5* and *KDM6* genes), has apparently no major impact on autosomal gene expression in myotubes*.* Further studies are needed to elucidate the mechanisms behind the sex-specific expression of autosomal genes and to have a basis for the development of female and male muscle models for *in vitro* studies. Future studies could further investigate whether sex hormones modulate DNA methylation in muscle cells *in vitro*, an area not addressed in the present work.

Our study has limitations. The initial study was not designed to explore sex differences, resulting in an unequal sex distribution. We addressed this with appropriate statistical modeling and focused analyses. Two female participants used contraceptives and one was postmenopausal, but none of them were identified as outliers in PCA analyses or showed a diverging pattern in expression data of analyzed genes and proteins. The menstrual cycle was not tracked, which may introduce additional hormonal variability, however, it reflects real-world diversity and enhances generalizability. Also, while we describe potential metabolic implications based on omics data, we did not directly measure metabolic fluxes. We found mostly DMRs on the X chromosome (7 in Chr X and 1 in Chr 2) corresponding to differential expression patterns conserved over 2 cohorts, but comparisons of monoallelic (male) and biallelic (female) DNA methylation levels of X-chromosomal CpG sites remain debatable [63]. We focused on overrepresentation analysis of significantly regulated transcripts and proteins to prioritize interpretability and stringency given the modest sample size, but gene set enrichment methods such as GSEA could provide additional insight. Furthermore, our cohort consisted of overweight or obese individuals with low cardiorespiratory fitness due to a sedentary lifestyle. It remains to be shown whether the results of the initial differences and in the adaptation to the 8 weeks of training can be translated to other cohorts differing in age, physical fitness and metabolic parameters.

In summary, we provide an in-depth multi-OMICs molecular characterization of female and male skeletal muscle. The pronounced sex-specific differences in transcripts and proteins mirror the functional differences in glucose and lipid metabolism and are deeply rooted in the epigenome.

Sex has also an impact on the response to exercise. Our data hint towards a potential protective effect of estrogen as we provide evidence on the transcriptional level that males experience higher oxidative and cellular stress in their muscles when cycling at the same individual intensity as females. The reduction in type 2 fiber-specific proteins specifically in males after only 8 weeks of training contributes to the endurance-trained proteomic profile in both sexes and underlines the high plasticity of skeletal muscle to adapt to repeated endurance exercise loads towards a metabolically beneficial phenotype independent of sex.

## CRediT authorship contribution statement

**Simon I. Dreher:** Writing – review & editing, Writing – original draft, Visualization, Validation, Project administration, Methodology, Investigation, Formal analysis, Conceptualization. **Thomas Goj:** Validation, Methodology, Investigation, Formal analysis, Data curation. **Christine von Toerne:** Writing – review & editing, Validation, Methodology, Formal analysis, Data curation. **Miriam Hoene:** Methodology, Investigation, Formal analysis. **Martin Irmler:** Methodology, Formal analysis, Data curation. **Meriem Ouni:** Writing – review & editing, Validation, Methodology, Investigation, Formal analysis. **Markus Jähnert:** Writing – review & editing, Validation, Methodology, Formal analysis. **Johannes Beckers:** Supervision, Resources. **Martin Hrabě de Angelis:** Supervision, Resources, Funding acquisition. **Andreas Peter:** Writing – review & editing, Supervision, Resources, Project administration, Funding acquisition. **Anja Moller:** Methodology, Investigation. **Andreas L. Birkenfeld:** Writing – review & editing, Supervision, Resources, Project administration, Funding acquisition. **Annette Schürmann:** Writing – review & editing, Supervision, Resources, Project administration, Methodology, Funding acquisition. **Stefanie M. Hauck:** Writing – review & editing, Supervision, Resources, Methodology. **Cora Weigert:** Writing – review & editing, Writing – original draft, Supervision, Resources, Project administration, Funding acquisition, Conceptualization.

## Disclosures

No conflicts of interest, financial or otherwise, are declared by the authors.

## Grants

This study was supported in part by grants from the German Federal Ministry of Education and Research (BMBF) to the German Centre for Diabetes Research (DZD e.V.) under Grant No. 01GI0925, 82DZD00302, 82DZD03D03), and the State of Brandenburg.

## Declaration of competing interest

The authors declare that they have no known competing financial interests or personal relationships that could have appeared to influence the work reported in this paper.

## Data Availability

Data will be made available on request.
