# Supplementary material for: Sex differences in resting skeletal muscle and the acute and long-term response to endurance exercise in individuals with overweight and obesity
Source: Mol Metab. 2025 Jun 16;98:102185. doi: 10.1016/j.molmet.2025.102185 (PMC12268009; doi:10.1016/j.molmet.2025.102185)
Supplement: Multimedia component 1 [file mmc1.docx]

# Sex differences in resting skeletal muscle and the acute and long-term response to endurance exercise in individuals with overweight and obesity

Simon I. Dreher^1^, Thomas Goj^1,2,3^, Christine von Toerne^4^, Miriam Hoene^1^, Martin Irmler^5^, Meriem Ouni^3,6^, Markus Jähnert^3,6^, Johannes Beckers^3,5,7^, Martin Hrabe de Angelis^3,5,7^, Andreas Peter^1,2,3^, Anja Moller^2,3,8^, Andreas L. Birkenfeld^2,3,8^, Annette Schürmann^3,6,9^, Stefanie M. Hauck^3,4^, Cora Weigert^1,2,3^

^1^Institute for Clinical Chemistry and Pathobiochemistry, Department for Diagnostic Laboratory Medicine, University Hospital Tübingen, Tübingen, Germany.

^2^Institute for Diabetes Research and Metabolic Diseases of the Helmholtz Zentrum München at the University of Tübingen, Tübingen, Germany.

^3^German Center for Diabetes Research (DZD e.V.), München-Neuherberg, Germany.

^4^Metabolomics and Proteomics Core, Helmholtz Center Munich, German Research Center for Environmental Health, 85764 Neuherberg, Germany

^5^Institute of Experimental Genetics, Helmholtz Zentrum München, Neuherberg, Germany ^6^Department of Experimental Diabetology, German Institute of Human Nutrition Potsdam-Rehbruecke (DIfE), Nuthetal, Germany, Potsdam, Germany.

^7^School of Life Sciences, Chair of Experimental Genetics, Technical University Munich, Freising 85764, Germany.

^8^Department of Internal Medicine IV, University Hospital Tübingen, Germany

^9^Institute of Nutritional Science, University of Potsdam, Brandenburg, Germany.

**Figure S 1 Timeline of the exercise intervention study**

**Figure S 2 Transcriptomic analysis of female vs. male skeletal muscle at baseline**

**Figure S 3 Transcriptomic and proteomic responses to acute exercise and training**

**Figure S 4 Conserved sex-specific differences in myotubes in vitro**

**Figure S 5 Sex hormone-specific transcriptional regulation in myotubes in vitro**

**Supplementary Table 1 Correlation of Transcription and Performance**

**Supplementary Data Table 1 Excel file containing epigenomic, transcriptomic and proteomic data analysis of the study**

##
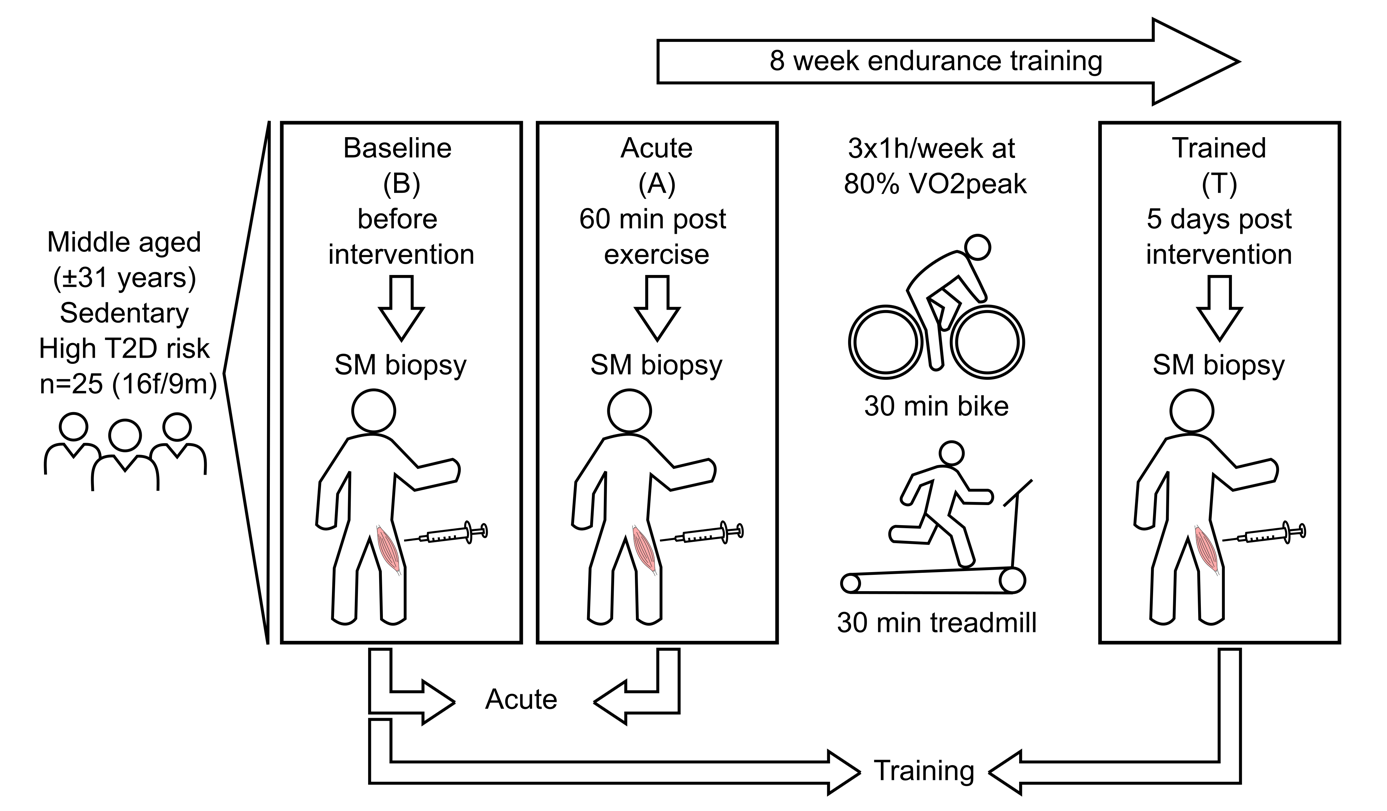


Figure S 1 Timeline of the exercise intervention study

Middle aged participants with a sedentary lifestyle and overweight or obesity (n=25, 16f/9m) underwent an 8-week supervised endurance exercise intervention consisting of 30min on ergometer and 30min on a treadmill at 80% VO2peak. Muscle biopsies were obtained at baseline (B) before exercise, 60min after the first acute ergometer exercise in an untrained state (A) and 5 days after the last exercise session after 8 weeks of training (T) in a rested state.


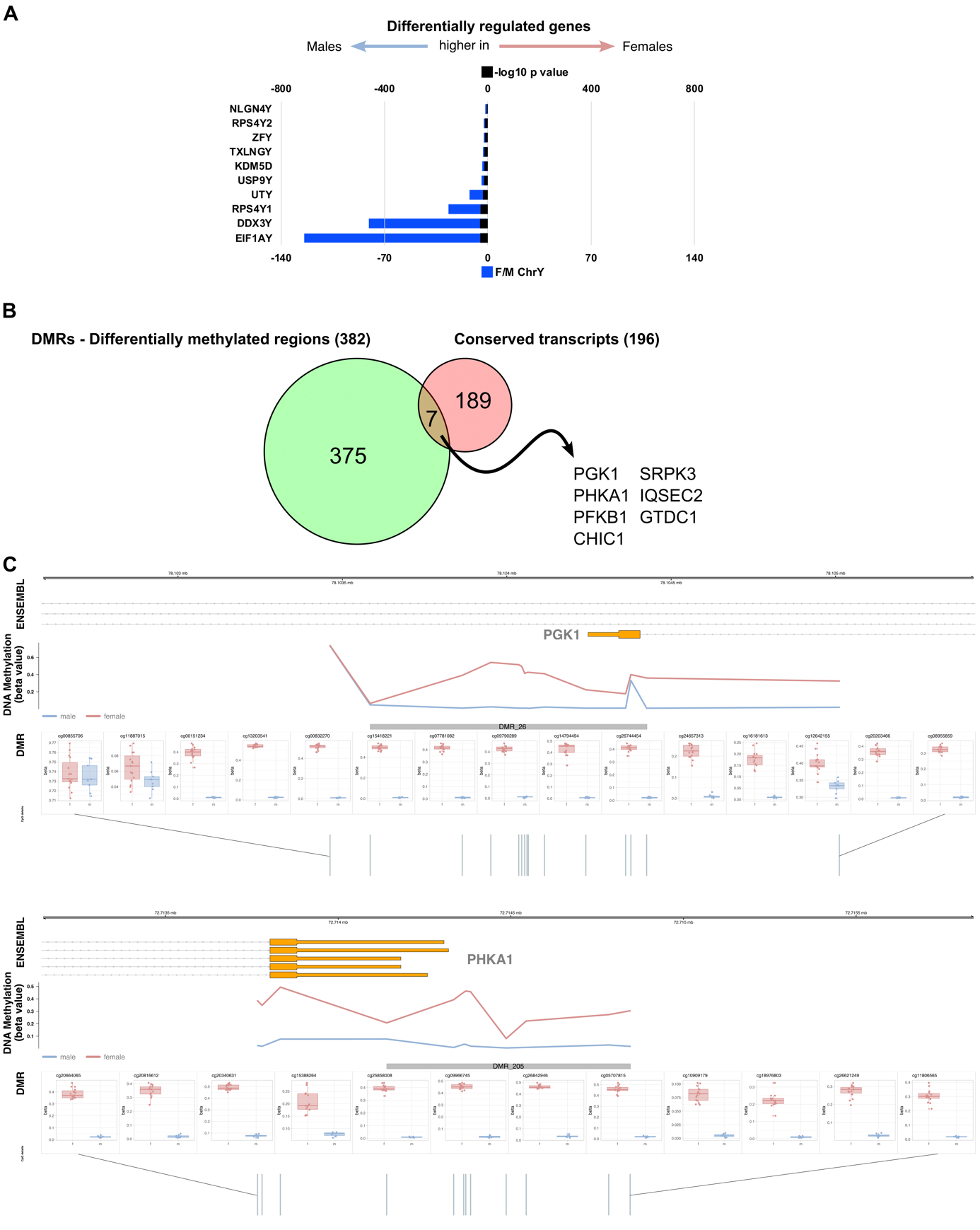


Figure S 2 Transcriptomic analysis of female vs. male skeletal muscle at baseline

Skeletal muscle biopsies obtained at baseline were analyzed for differentially methylated regions (DMRs) and transcriptomic differences between females and males. A) Top 10 transcripts higher expressed in males (left) and females (right) conserved in this and another independent cohort [1] are plotted, fold change (F/M) in A) Y-chromosomal (blue) genes (bottom axis), -log10 p-values are plotted in black (top axis). Statistical significance was determined by limma t-test Bayes p < 0.05, n=25 (16f/9m) and validation cohort (n=19; 12f/6m). B) Overlap between identified DMRs and differentially expressed transcripts conserved over both cohorts. C) Detailed view of CpG sites in DMR 26 and 205 associated with genes PGK1 and PHKA1.


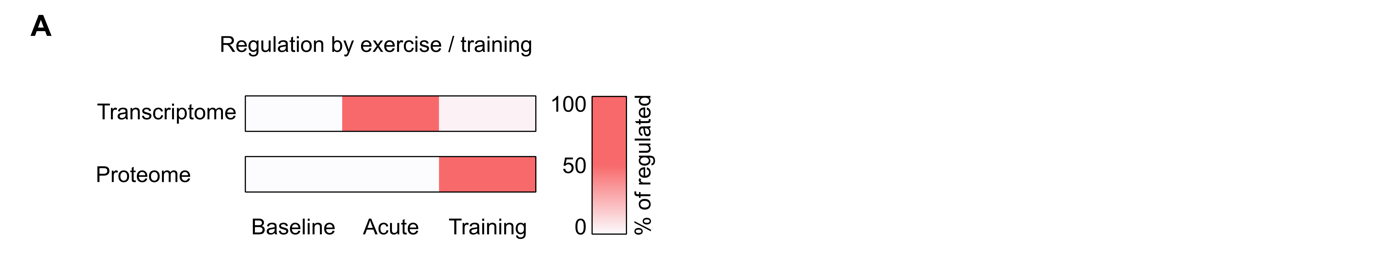


Figure S 3 Transcriptomic and proteomic responses to acute exercise and training

A) Heatmap depicting the percentage of all transcripts or proteins regulated by acute exercise and training. Out of all transcripts regulated by exercise and training, 98% were regulated in response to acute exercise and 4% after training. Out of all proteins regulated by exercise and training, 1% were regulated in response to acute exercise and 100% after training. Statistical significance was determined by limma t-test Bayes BH, p < 0.05, n=25 (16f/9m).


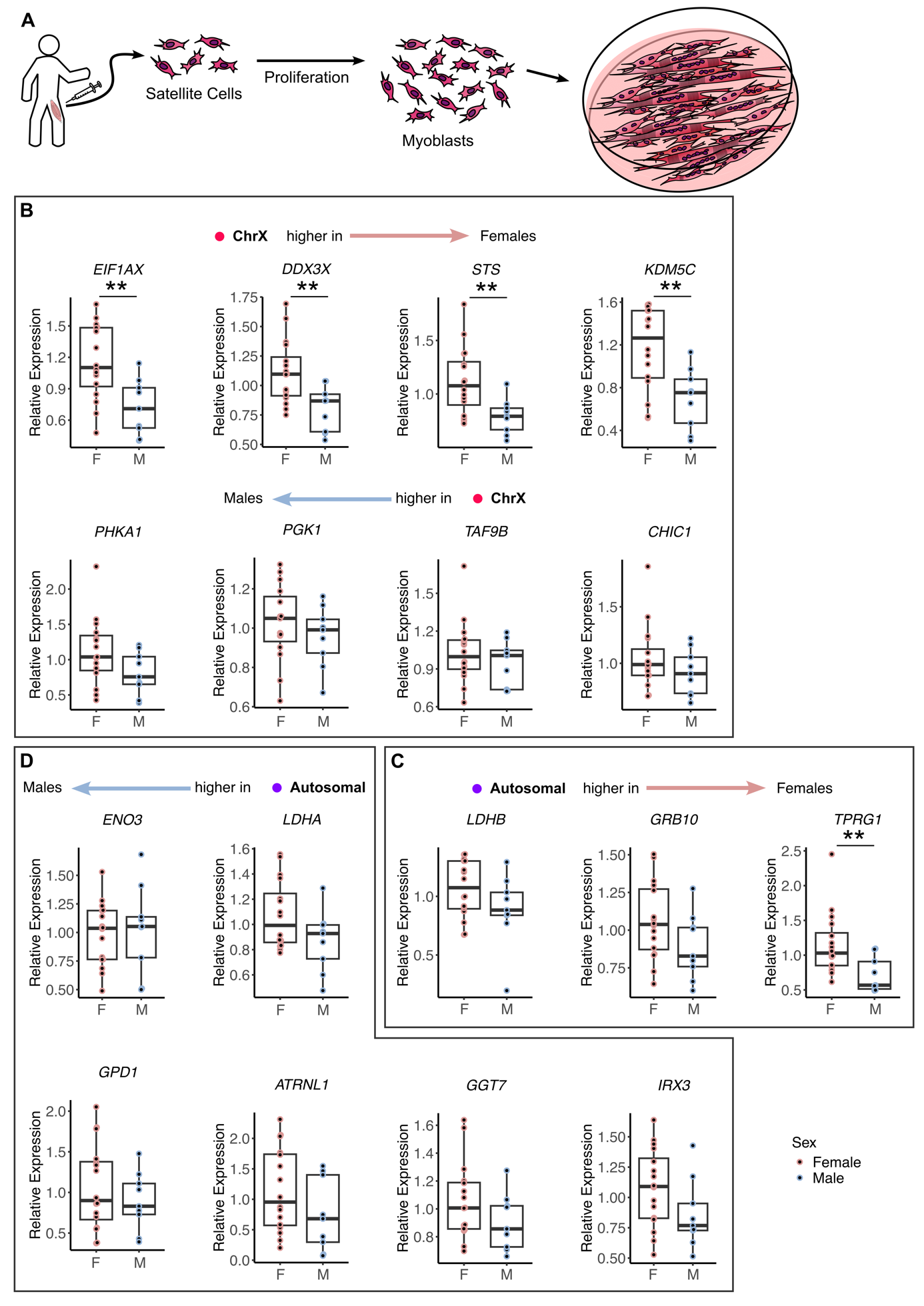


Figure S 4 Conserved sex-specific differences in myotubes in vitro

A) Satellite cells were isolated from skeletal muscle biopsies, myoblasts were cultured and differentiated into myotubes for 7 days. Expression of B) X-chromosomal and C/D) autosomal genes is shown for female (F) and male (M) myotubes. Arrows indicate sex-specific differential expression *in vivo* in skeletal muscle biopsies. Significant differences were assessed using one-way ANOVA LSD, ** p<0.01, *** p<0.001, n=25 (16f/9m).


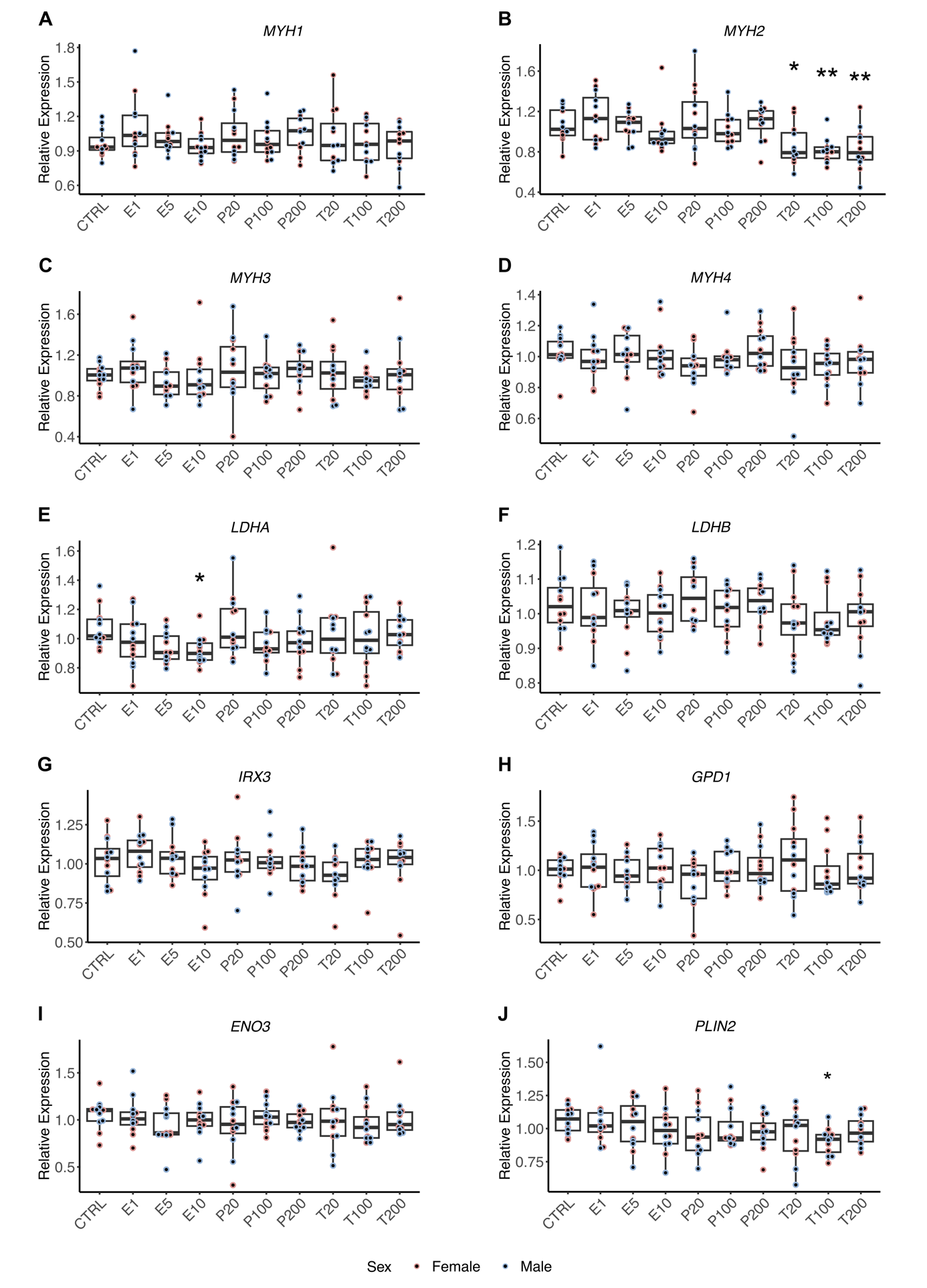


Figure S 5 Sex hormone-specific transcriptional regulation in myotubes in vitro

A) Myoblasts derived from 6 female and 6 male donors were cultured and differentiated into myotubes for 7 days. During differentiation, myotubes were treated with estradiol (E) (1/5/10nM), progesterone (P) (20/100/200nM) or testosterone (T) (20/100/200nM) or left untreated (CTRL). Expression of mRNA was measured for A) *MYH1*, B) *MYH2*, C) *MYH3*, D) *MYH4*, E) *LDHA*, F) *LDHB*, G) *IRX3*, H) *GPD1*, I) *ENO3*, J) *PLIN2* in myotubes after hormonal treatment, respectively. Statistical significance was determined by using one-way ANOVA Bonferroni, n=12 (6f/6m). Red dots, female donor; blue dots; male donor.

Supplementary Table 1 Correlation of Transcription and Performance

|  | **Pearson Correlation with Performance [W]** | |
| --- | --- | --- |
|  | **r** | **p-Value** |
| ***ATF3*** | -0.040 | 0.848 |
| ***JUN*** | 0.053 | 0.800 |
| ***STK39*** | 0.246 | 0.235 |
| ***SLC39A14*** | 0.235 | 0.258 |
| ***HMOX*** | 0.234 | 0.259 |
| ***MT1A*** | 0.119 | 0.570 |
| ***MT1B*** | 0.022 | 0.916 |

Supplementary Data Table 1 Excel file containing epigenomic, transcriptomic and proteomic data analysis of the study

Supplementary Data Table 1 can be accessed via figshare DOI: 10.6084/m9.figshare.28918622
